# Integrated bioinformatics analysis identifies an immune-related gene signature associated with non-obstructive azoospermia

**DOI:** 10.1371/journal.pone.0360305

**Published:** 2026-10-12

**Authors:** Yucheng Zhong, Songqing Ye, Hongshen Lai, Xiangyu Zeng, Ge Song, Xiaofu Qiu

**Affiliations:** 1 Department of Urology, The Affiliated Guangdong Second Provincial General Hospital of Jinan University, Guangzhou, China; 2 Assisted Reproductive Technology Center, The Affiliated Foshan Women and Children Hospital, Guangdong Medical University, Foshan, China; 3 Department of Urology, Affiliated Hospital Group of Guangdong Medical University Shenzhen Baoan Central Hospital, Shenzhen, Guangdong, China; University of California San Diego, UNITED STATES OF AMERICA

## Abstract

Non-obstructive azoospermia (NOA) is a severe male infertility disorder. This study aims to identify immune-related biomarkers for explore immune-related molecular signatures associated with NOA. Here, we integrated transcriptomic data from four independent cohorts (GSE145467, GSE45885, GSE45887, GSE9210) using an integrative pipeline combining differential expression analysis, weighted gene co-expression network analysis (WGCNA), and consensus-based multi-machine learning to identify an immune-related gene signature associated with NOA. We identified 16 immune-related differentially expressed genes through WGCNA, which were refined into a 12-gene panel—*Hub NOA.Sig* (*CD14, CD19, CMTM2, CXCL16, HSPA1L, HSPA2, IGF1, IL13, POMC, RBP1, STAB2, TYROBP*)—using five feature selection algorithms (Bagged Trees, Bayesian, Boruta, LASSO, LVQ). A Random Forest classifier based on *Hub NOA.Sig* yielded good classification performance with an AUC of 0.87. This signature offers a transcriptomic reference for characterizing NOA heterogeneity and exploring immune-related molecular features. Single-sample gene set enrichment analysis (ssGSEA) revealed significant alterations in six immune cell populations—including effector memory CD4 ⁺ /CD8 ⁺ T cells, regulatory T cells, and plasmacytoid dendritic cells—and demonstrated strong correlations between hub genes and specific immune infiltrates. Furthermore, drug-gene interaction screening and molecular docking predicted loperamide-*POMC*, acitretin-*RBP1*, and retinol-*RBP1* as candidate drug-gene pairs for subsequent experimental validation. This work provides transcriptomic clues and candidate molecular targets for future mechanistic investigations into NOA-related male infertility.

## 1. Introduction

Non-obstructive azoospermia (NOA) represents one of the most challenging forms of male infertility, affecting approximately 1% of men and accounting for 10–20% of male infertility cases. It is defined by the absence of spermatozoa in the ejaculate due to intrinsic testicular failure rather than physical obstruction [1,2]. Current diagnosis relies on clinical evaluation, hormonal profiling, and invasive testicular biopsy, which provide limited insight into underlying molecular mechanisms and exhibit poor predictive value for assisted reproductive technology (ART) outcomes such as testicular sperm extraction followed by intracytoplasmic sperm injection (TESE-ICSI). Despite advances in ART, success rates remain suboptimal, and effective pharmacological interventions are lacking [3,4].

The testis maintains immune privilege through structural barriers (e.g., blood-testis barrier), local immunosuppressive mediators (e.g., TGF-β, IL-10), and specialized immune cell populations, thereby protecting autoantigenic germ cells from immune attack [5,6]. However, this tolerance can be compromised by infection, trauma, or autoimmune triggers, leading to chronic inflammation, immune cell infiltration, and subsequent germ cell apoptosis—a process increasingly implicated in NOA. While isolated cytokines such as TNF-α and IL-6 have been associated with NOA, comprehensive characterization of the testicular immunogenomic landscape—and its functional impact on spermatogenesis—remains limited [7,8].

Recent advances in high-throughput genomics and computational biology offer unprecedented opportunities to dissect disease-specific pathways. Weighted gene co-expression network analysis (WGCNA) enables identification of functionally coordinated gene modules, while machine learning approaches enhance biomarker discovery by minimizing overfitting and improving generalizability [9–11]. Moreover, databases such as ImmPort provide curated collections of immune-related genes, facilitating focused interrogation of immunogenomic landscape [12]. Despite these methodological advances, prior NOA studies have often been limited by small sample sizes, single-cohort datasets, or reliance on singular computational approaches, leading to inconsistent or non-generalizable findings [13,14]. Furthermore, the translational potential of discovered biomarkers is constrained without multi-center validation and exploration of drug-gene interactions.

In this study, we leveraged multi-cohort transcriptomic data from the Gene Expression Omnibus (GEO) database and applied an integrative pipeline combining differential expression analysis, WGCNA, and a consensus-driven multi-machine learning framework to identify a robust immune-derived gene signature for NOA. We define this 12-gene panel as the Hub Immune Signature for NOA (*Hub NOA.Sig*). This immune-related signature enables transcriptomic classification, immune landscape characterization, molecular subtyping and computational drug prediction, providing a valuable reference for further mechanistic and translational research on NOA. Therefore, there is an urgent need for a systems-level, multi-cohort validated immune signature that not only improves diagnosis but also informs underlying biology and reveals druggable targets.

## 2. Materials and methods

### 2.1. Data download

To develop an immune-based predictive model for NOA for clinical precision medicine, we downloaded four datasets from the GEO database [15] (https://www.ncbi.nlm.nih.gov/geo): GSE145467 [16] (Homo sapiens, GPL4133, including 10 NOA patients and 10 obstructive azoospermia patients’ testis samples); GSE45885 [17] (Homo sapiens, GPL6244, including 27 NOA and 4 obstructive azoospermia patients’ testis samples); GSE45887 [18](Homo sapiens, GPL6244, including 16 NOA and 4 obstructive azoospermia patients’ testis samples); GSE9210 [19] (Homo sapiens, GPL887, including 47 NOA and 11 obstructive azoospermia patients’ testis samples). In our research, NOA samples were treated as the disease group, while obstructive azoospermia samples were used as controls.

We obtained 2,483 immune-related genes from the ImmPort database (https://www.immport.org/home) for subsequent analysis, as detailed in S1 Table. Additionally, we identified four sets of molecular markers for predicting NOA from previous studies for model comparison purposes. The data were accessed on December 15, 2025. All datasets used in this study are fully anonymized and publicly available; the authors had no access to any identifying information about individual participants.

### 2.2. Identification of immune-related differentially expressed genes

We utilized the “limma” package [20] to identify genes with differential expression (adjusted *P* ≤ 0.05) between NOA testis samples and OA control samples, extracting genes that exhibited differential expression in at least two datasets as candidate genes. Subsequently, we filtered the candidate genes to select immune-related genes for constructing the predictive model. Differential expression analysis was performed using the limma package, with the Benjamini–Hochberg method adopted for multiple testing correction to control the false discovery rate.

We analyzed each GEO dataset independently for differential expression without applying additional batch correction or cross-platform normalization. To minimize the influence of platform heterogeneity and latent batch effects, we retained only genes with consistent differential expression in at least two datasets. This consensus-based strategy was used to filter stable, reproducible molecular signatures rather than relying on formal batch adjustment algorithms.

### 2.3. Weighted gene co-expression network analyses

Based on the GSE145467 dataset and immune-related differentially expressed genes, we constructed a gene co-expression network using the R package “WGCNA” [21]. First, we calculated the correlation coefficient between any two genes and applied a weighted value of the correlation coefficients to form a scale-free network. Then, we constructed a hierarchical clustering tree based on gene correlations. Different branches of the clustering tree represent distinct gene modules, with different colors indicating different modules, followed by calculating module significance. In this study, WGCNA utilized genes with expression variance in the top 75% among all genes from the GSE145467 dataset as input, setting the minimum module gene number to 50 and softpower to 5 (the optimal power threshold). Modules with P values less than or equal to 0.05 were selected for further analysis.

### 2.4. Multi-machine learning screening of immune-related core biomarkers

To further identify core signatures from the 16 immune-related DEGs (NOA.sig) derived from WGCNA, we adopted the GSE145467 dataset as the training set and integrated five machine learning algorithms for feature selection. To avoid bias from a single algorithm, genes detected by at least two algorithms were finally defined as key molecular markers (*Hub NOA.Sig*). All analyses were performed in R 4.3.1, mainly using the packages glmnet, caret, Boruta, and klaR.

LASSO regression was implemented via the glmnet package. To reduce randomness from a single cross‑validation, we performed 1000 iterations of LASSO feature selection. In each iteration, five‑fold cross‑validation (cv.glmnet) was applied to fit a binary logistic model with family = “binomial”. The penalty parameter lambda.min with the minimum cross‑validation error was selected, and genes with non‑zero coefficients were extracted. The occurrence frequency of each gene across all iterations was counted, and the most frequent gene combination was retained as the LASSO-derived feature set.

The Boruta algorithm was performed using the Boruta package based on random forest. It compared the importance Z‑value of each original feature with permuted shadow features. We set doTrace = 3 to display the iteration process. After tentative feature confirmation via TentativeRoughFix, definitive important genes were extracted using getSelectedAttributes with withTentative = FALSE.

Learning Vector Quantization (LVQ) was implemented using the train function in the caret package. Data standardization was conducted before model training with preProcess = “scale”. The resampling scheme adopted repeated five‑fold cross‑validation (method = “repeatedcv”, number = 5). The hyperparameter tuning grid was set as size = 6, k = 1:2. Variable importance was calculated with the varImp function, and genes with an importance score > 0.5 were regarded as key features selected by LVQ.

Treebag and Bayesian algorithms were both implemented under the recursive feature elimination (RFE) framework via the rfe function in the caret package. For Treebag, treebagFuncs was specified with three‑fold cross‑validation (method = “cv”, number = 3) for feature reduction. For the Bayesian algorithm, the naive Bayes function nbFuncs was used, and feature subsets ranging from 2 to the total gene number were evaluated under three‑fold cross‑validation. The optimal feature subset was finally extracted using the predictors function for both algorithms.

Gene lists screened by the five algorithms (LASSO, Boruta, LVQ, Treebag, Bayesian) were integrated, and intersections among algorithms were visualized by an Upset plot. Genes identified by no fewer than two algorithms were retained as *Hub NOA.Sig* for subsequent diagnostic model construction and validation.

### 2.5. Construction of the non-obstructive azoospermia predictive model

We trained the NOA predictive model using *Hub NOA.Sig*, employing five machine learning (ML) algorithms for benchmarking, including Support Vector Machine (SVM), Naive Bayes (NB), Random Forest (RF), K-Nearest Neighbors (KNN), and LogitBoost algorithms, utilizing five-fold cross-validation (CV) to construct the training set model. To ensure robustness, we repeated the optimization process ten times using different random seeds for each single resampling. To establish the predictive model for NOA, the performance of candidate classifiers was assessed on an independent validation set, and the optimal classifier was selected based on superior classification accuracy. To comprehensively evaluate the stability of classification performance of each machine learning classifier, we performed 50 rounds of repeated five-fold cross-validation for all five algorithms. The mean AUC, standard deviation (SD_AUC) and 95% confidence interval (CI95) of each model were calculated based on the cross-validation results for quantitative stability assessment. All relevant statistical indicators were summarized into a dedicated supplementary table for clear presentation without cluttering the main text.

### 2.6. Comparison of *Hub NOA.Sig* with other signatures

To further evaluate the predictive value of *Hub NOA.Sig*, we compared it with other reported predictive biomarkers for NOA, including studies by Zhong et al. 2022 [22]; Dong et al. 2021 [23]; He et al. 2021 [24]; Omolaoye et al. 2022 [25]. We compared the predictive capabilities of other biological markers with *Hub NOA.Sig* in GSE145467, GSE45885, GSE45887, and GSE9210 regarding NOA prediction performance.

### 2.7. Single-sample gene set enrichment analysis

ssGSEA is a method used to assess the relationship between individual sample gene expression patterns and predefined gene sets. In immune infiltration analysis, ssGSEA could evaluate the expression levels of immune-related genes in a biological sample to infer the abundance and activity of immune cell types. Immune cells include various human immune cell subtypes: CD8^+^ T cells, dendritic cells, macrophages, regulatory T cells, etc. We calculated enrichment scores using the ssGSEA algorithm from the R package GSVA to represent the infiltration levels of different types of immune cells in each sample.

### 2.8. Drug sensitivity prediction

We explored the interactions between *Hub NOA.Sig* genes and small molecules using the Drug-Gene Interaction Database (DGIdb; https://dgidb.org/) and CMap (Connectivity Map; https://clue.io/) to identify potential drug targets and therapeutic drugs for NOA. The DGIdb database is a drug-gene interaction database that provides information on the relationships between genes and their known or potential drug associations. It contains over 14,000 drug-gene interactions involving 2,600 genes and 6,300 drugs targeting these genes, along with 6,700 other genes that might become drug targets in the future. The Connectivity Map is a gene expression database constructed by researchers from Harvard, Cambridge University, and MIT, establishing a biological application database that correlates perturbations (including small molecules) with gene expression differences in human cells. By integrating these two databases, we could identify the interaction details of *Hub NOA.Sig* with small molecules and discover potential drugs significantly associated with NOA, facilitating its treatment.

### 2.9. Molecular docking

Through drug sensitivity prediction, we identified potential drugs significantly associated with NOA. We downloaded the molecular structures of the drugs from the PubChem database (https://pubchem.ncbi.nlm.nih.gov/). The corresponding target protein molecular structures of potential drugs were obtained from the Protein Data Bank (PDB; http://www.rcsb.org/). We utilized CB-Dock2 (https://NOAd.labshare.cn/cb-dock2/php/index.php) to visualize the docking of potential drugs with their targets. PubChem is the world’s largest free chemical information database, enabling searches for chemicals by name, molecular formula, structure, and other identifiers, providing information on chemical and physical properties, biological activity, safety and toxicity, patents, literature citations, etc. The PDB is a database established in 1971 by the Brookhaven National Laboratory of the United States and maintained by the Research Collaboratory for Structural Bioinformatics (RCSB). It is the primary database for collecting 2.5D structures of biological macromolecules (proteins, nucleic acids, and carbohydrates), determined through experimental techniques such as X-ray diffraction, nuclear magnetic resonance, and electron diffraction. CB-Dock2 is an improved version of the CB-Dock server for protein-ligand blind docking, integrating cavity detection, docking, and homology template fitting. With the 3D structures of the proteins and ligands, we could predict their binding sites and affinities, facilitating computer-aided drug discovery.

### 2.10. Statistical analysis

All statistical analyses were conducted using R software (version 4.3.1). The Wilcoxon rank-sum test was used to compare differences between two groups. The Kruskal-Wallis test was applied to evaluate differences among more than two groups. Correlation analyses were performed using the Spearman correlation method. A *P* value of < 0.05 was considered statistically significant.

## 3. Results

### 3.1. Study workflow and analytical pipeline

The overall analytical strategy of this study is illustrated in Fig 1. Briefly, four independent transcriptomic datasets were downloaded from the GEO database, with obstructive azoospermia samples included as controls. Differential expression analysis was performed using the limma package across all cohorts. Genes differentially expressed in at least two datasets were intersected with immune-related genes from the ImmPort database, yielding 44 IRDEGs. WGCNA was subsequently performed based on the GSE145467 dataset to identify immune gene modules significantly correlated with NOA status. The intersection of IRDEGs and key module genes generated 16 candidate genes.

**Fig 1:**
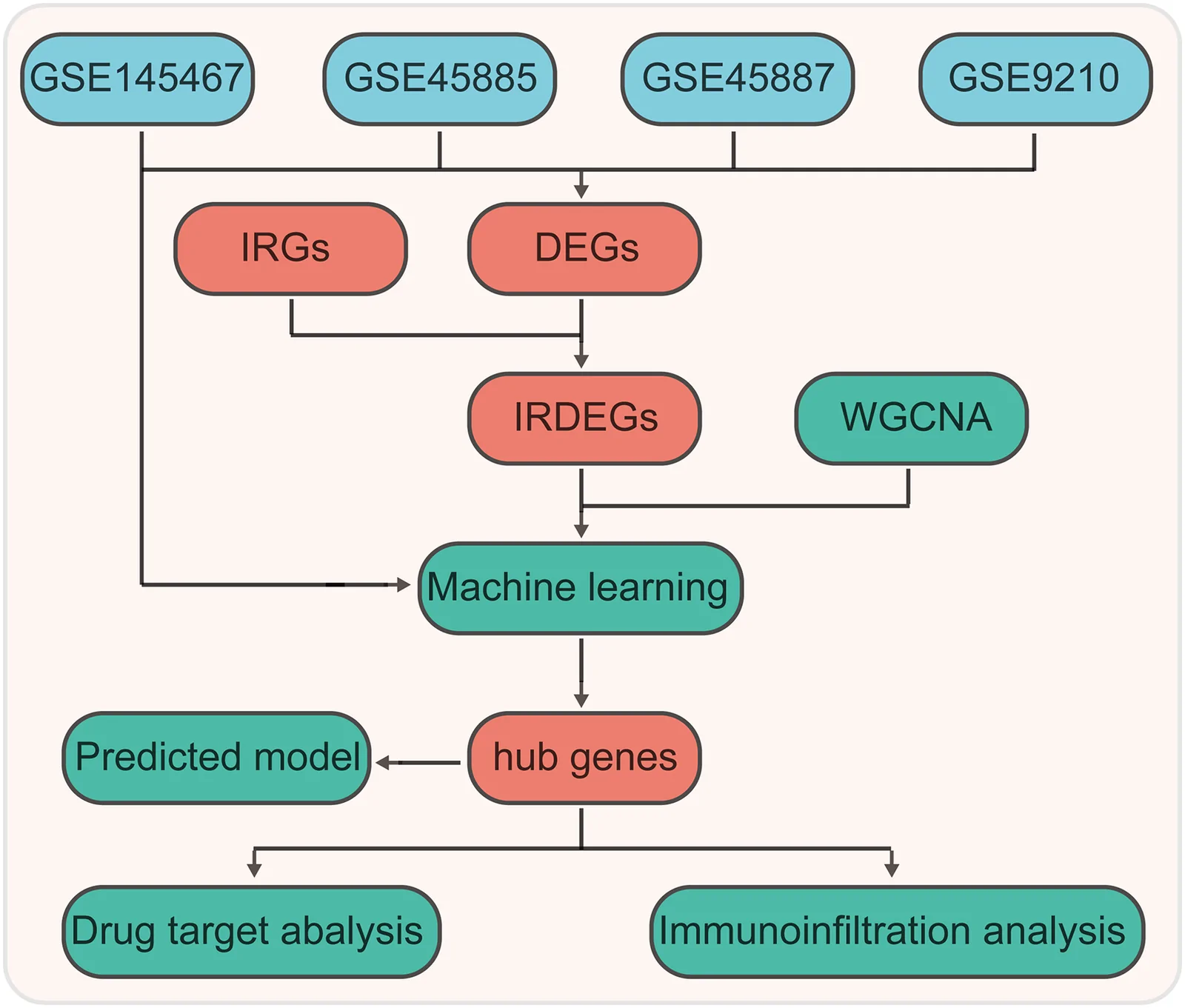
Study workflow and integrative analytical pipeline. Schematic overview of the multi-step framework used to identify and validate *Hub NOA.Sig*. Transcriptomic datasets (GSE145467, GSE45885, GSE45887, GSE9210) were downloaded from the GEO. DEGs were identified using the limma package, followed by intersection with immune-related genes from the ImmPort database to yield IRDEGs. WGCNA was performed on GSE145467 to identify modules correlated with NOA. Consensus feature selection was conducted using five machine learning algorithms (LASSO, Boruta, Bayesian, LVQ, Bagged Trees) to derive the final 12-gene signature (*Hub NOA.Sig*). The signature was evaluated for diagnostic performance, immune infiltration patterns via ssGSEA, and drug repurposing potential through DGIdb and molecular docking. IRGs: immune-related genes. DEGs: differentially expressed genes. IRDEGs: immune-related differentially expressed genes. WGCNA: weighted gene co-expression network analyses.

Five machine learning algorithms, including Bagged Trees, Bayesian, Boruta, LASSO, and LVQ, were applied for feature selection. Genes detected by no fewer than two algorithms were retained to construct the final 12-gene signature *Hub NOA.Sig*. Multiple classifiers were established and compared, and the Random Forest model with the best performance was selected as the final diagnostic model. In addition, ssGSEA was used to profile the immune infiltration landscape, and drug–gene interaction analysis combined with molecular docking was conducted to explore potential candidates for drug repurposing.

### 3.2. Discovery of immune-related gene modules via weighted gene co-expression network analyses

To identify potential immune-related biomarkers from NOA patient data collected from the GEO public database, we first screened for differentially expressed genes. Utilizing the “limma” R package, we conducted differential analysis across four datasets containing NOA patients and OA control data, identifying differentially expressed genes in each dataset and filtering for those expressed differentially in at least two datasets for further analysis. The results indicated that a total of 208 genes exhibited differential expression in the GSE145467 dataset, 1,602 genes in GSE45885, 70 genes in GSE45887, and 9,111 genes in GSE9210. Detailed differential analysis results are shown in S2–S5 Tables. As illustrated in Fig 2A-D, a total of 1,793 genes exhibited differential expression in at least two datasets. We further filtered the 1,793 genes to select immune genes obtained from the ImmPort database, resulting in 44 immune-related differentially expressed genes (NOA.sig) (Fig 2E).

**Fig 2:**
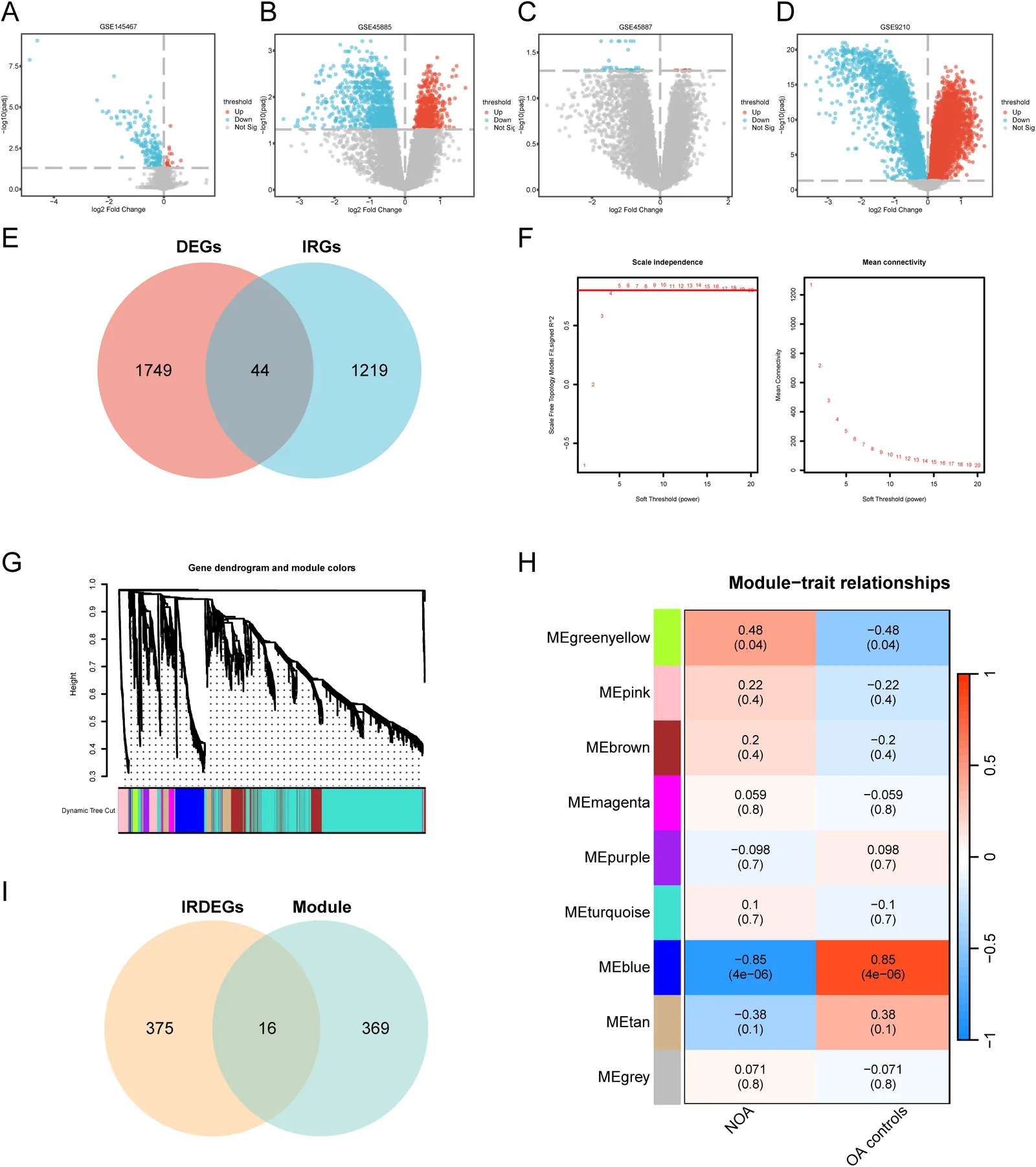
Identification of immune-related differentially expressed genes and WGCNA-derived modules. (A–D) Volcano plots showing DEGs in four independent cohorts: (A) GSE145467, (B) GSE45885, (C) GSE45887, and (D) GSE9210 (|log_2_FC| > 0.5, adjusted *P* < 0.05). Red dots represent upregulated genes; blue dots represent downregulated genes. (E) Venn diagram illustrating the intersection of DEGs across at least two datasets and subsequent filtering for immune-related genes from ImmPort, yielding 44 IRDEGs. (F) Scale-free topology fit index plot used to determine soft-threshold power (β = 5). (G) Hierarchical clustering dendrogram of co-expression modules identified by WGCNA (each color represents a module; gray = unassigned). (H) Module-trait relationship heatmap showing correlation between eigengenes and NOA status (*P* < 0.05). (I) Intersection of IRDEGs and WGCNA module genes resulting in 16 candidate genes.

The identification of key immune-related genes aids the further development of immune-based predictive biomarkers for NOA. To identify critical genes, we constructed a WGCNA for immune-related NOA-associated genes. In the scale-free network, the soft threshold was set to 5 (Fig 2F). Following this, a scale-free network was constructed based on the soft threshold, and a topological matrix was generated for hierarchical clustering. The minimum module size was set to 50 genes. We calculated the characteristic genes of each module and merged similar modules. Ultimately, we identified seven modules, with the gray module representing unassigned genes (Fig 2G). Among the eigengenes of the nine modules, two exhibited statistically significant differences (Fig 2H). The intersection of IRDEGs and module genes yielded 16 NOA.sig genes (*IGF1*, *PTN*, *HLA-DRB5*, *TYROBP*, *RBP1*, *CXCL16*, *HSPA1L*, *CD14*, *HSPA2*, *TNFSF13*, *IL13*, *STAB2*, *CMTM2*, *CD19*, *POMC*, *HLA-DRB1*), as shown in Fig 2I.

### 3.3. Consensus selection of hub genes using five machine learning algorithms

To further screen for core immune-related key genes (*Hub NOA.Sig*) and explore their relationship with NOA occurrence, this study analyzed the relationship between NOA.sig and NOA occurrence using five machine learning algorithms: Bagged Trees, Bayesian, Wrapper (Boruta), LASSO, and LVQ. In the Bagged Trees algorithm, four NOA.sig genes (*CXCL16*, *HSPA1L*, *IL13*, *IGF1*) closely related to NOA occurrence were identified, as depicted in Fig 3A. The Bayesian algorithm yielded three key genes (*CXCL16*, *HSPA1L*, *CMTM2*), with their accuracy distribution shown in Fig 3B. The Boruta algorithm identified ten key genes (*IGF1*, *TYROBP*, *CXCL16*, *HSPA1L*, *HSPA2*, *IL13*, *STAB2*, *CMTM2*, *CD19*, *POMC*) with importance values illustrated in Fig 3C. The LASSO algorithm identified six key genes (*CD14*, *CXCL16*, *HSPA1L*, *HSPA2*, *IL13*, RBP1), shown in Fig 3D. Finally, the LVQ algorithm identified 16 key genes (*IGF1*, *PTN*, *HLA_DRB5*, *TYROBP*, *RBP1*, *CXCL16*, *HSPA1L*, *CD14*, *HSPA2*, *TNFSF13*, *IL13*, *STAB2*, *CMTM2*, *CD19*, *POMC*, *HLA_DRB1*), as depicted in Fig 3E.

**Fig 3:**
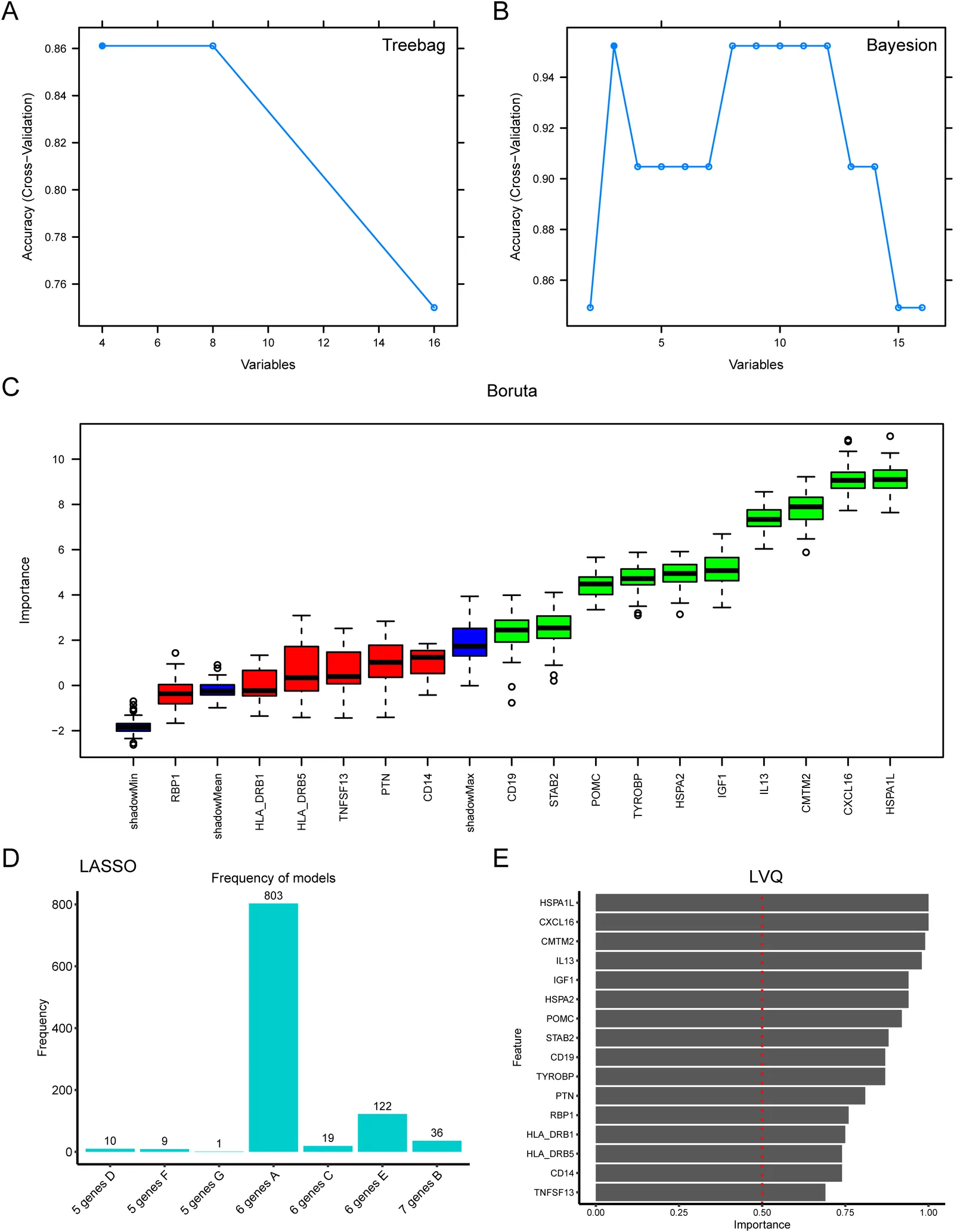
Multi-machine learning consensus identifies *Hub NOA.* *Sig* genes. Bar plots or radar charts depicting gene importance scores derived from five machine learning algorithms: (A) Bagged Trees, (B) Bayesian algorithm, (C) Boruta, (D) LASSO regression, and (E) LVQ. Genes selected by each algorithm are highlighted. Only genes appearing in ≥2 algorithms were retained for final signature construction.

### 3.4. Development and validation of a high-performance diagnostic classifier

To create a stable and high-performing predictive model, we selected key genes of NOA.sig that were present in at least two algorithms as immune-related key genes for predicting NOA occurrence (Fig 4A). The results indicated that 12 core immune-related key genes (*Hub NOA.Sig*) were most closely associated with NOA events: *CD14, CD19, CMTM2, CXCL16, HSPA1L, HSPA2, IGF1, IL13, POMC, RBP1, STAB2, TYROBP.* The chromosomal locations of these 12 *Hub NOA.Sig* genes are illustrated in Fig 4B, showing distribution across chromosomes 2, 3, 5, 6, 12, 14, 16, 17, and 19.

**Fig 4:**
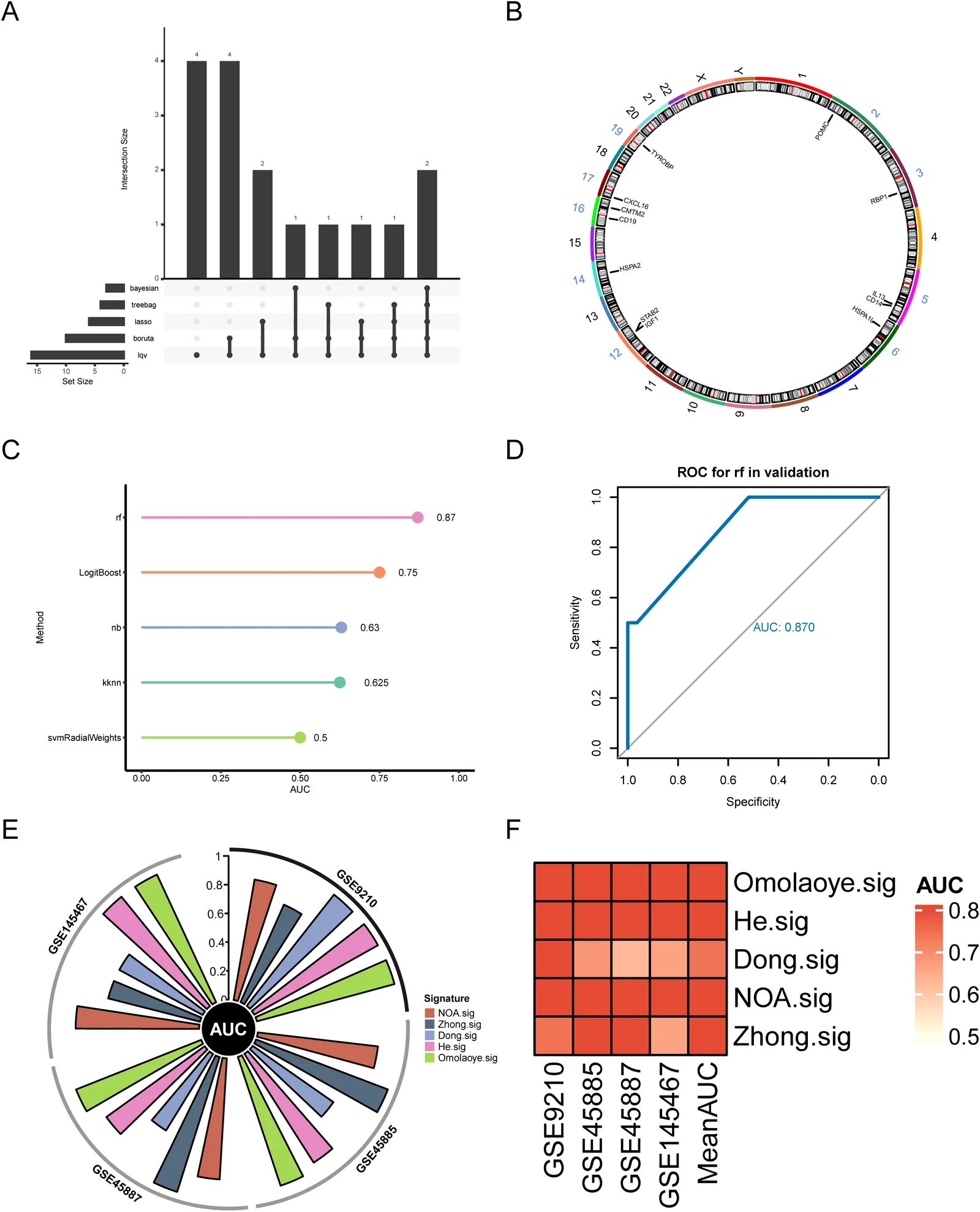
Diagnostic performance and cross-validation of *Hub NOA.* *Sig.* (A) Overlap of selected genes across five ML algorithms, leading to the final 12-gene *Hub NOA.Sig*. (B) Chromosomal localization of *Hub NOA.Sig* genes. (C) ROC curves comparing classification performance of five machine learning models in the validation cohort; Random Forest achieved the highest AUC (0.87). (D) Boxplot of AUC values from 10 repetitions of 5-fold cross-validation for Random Forest model. (E) Performance comparison of *Hub NOA.Sig* versus previously published signatures across four datasets (GSE145467, GSE45885, GSE45887, GSE9210). (F) Mean AUC (±SD) across all datasets demonstrating superior stability and accuracy of *Hub NOA.Sig*.

To study the relationship between *Hub NOA.Sig* and NOA events, we collected datasets related to NOA events from the GEO database, including GSE145467, GSE45885, GSE45887, and GSE9210. We preprocessed and organized the samples in these cohorts for analysis. We built predictive models for NOA events in the training set using five algorithms, selecting the best predictive model by comparing the AUCs of different algorithm models, and performed 10 repetitions of five-fold cross-validation for parameter optimization. We evaluated and compared these models’ AUCs in the validation cohort, finding that the predictive model constructed using the Random Forest algorithm exhibited superior diagnostic capability compared to other algorithms, with an AUC of 0.87 (Fig 4C). The standard deviations and 95% confidence intervals of AUC values derived from 50 repeated cross-validation for all five classifiers are summarized in S6 Table. The AUC results from the Random Forest model are specifically shown in Fig 4D. To validate the predictive advantages of *Hub NOA.Sig* for NOA events, we compared its predictive performance with previously published predictive models. Results demonstrated that *Hub NOA.Sig* exhibited high and stable predictive performance across GSE145467, GSE45885, GSE45887, and GSE9210 (Fig 4E). Fig 4F illustrates the performance of different predictive features for NOA events across various datasets, where MeanAUC represents the average AUC across the four datasets, indicating the comprehensive ability of *Hub NOA.Sig* to predict NOA events with high accuracy and stability.

### 3.5. Immune microenvironment remodeling in non-obstructive azoospermia patients

We assessed the distribution of 28 immune cell types through ssGSEA and compared the abundance of different immune cell distributions between the NOA and OA control groups using the Wilcoxon test (Fig 5A). The results indicated significant differences in the distribution of Effector memory CD4^+^ T cells, Effector memory CD8^+^ T cells, Regulatory T cells, Activated dendritic cells, Natural killer T cells, and Plasmacytoid dendritic cells between the groups (*P* < 0.05). Through correlation analysis of *Hub NOA.Sig* with the distribution of various immune cells, we found a strong correlation between the abundance of immune cell infiltration (Effector memory CD4^+^ T cells, Effector memory CD8^+^ T cells, Regulatory T cells, Activated dendritic cells, Natural killer T cells, Plasmacytoid dendritic cells) and *Hub NOA.Sig* (Fig 5B). Notably, *IGF1* showed a significant positive correlation with Plasmacytoid dendritic cells (Fig 5C) (r = 0.765, *P* = 8.52e-05) and with Regulatory T cells (Fig 5D) (r = 0.794, *P* = 2.87e-05). Conversely, *HSPA1L* exhibited a significant negative correlation with Effector memory CD4^+^ T cells (Fig 5E) (r = −0.695, *P* = 0.000665), while *CD19* showed a significant negative correlation with Activated dendritic cells (Fig 5F) (r = −0.595, *P* = 0.00561).

**Fig 5:**
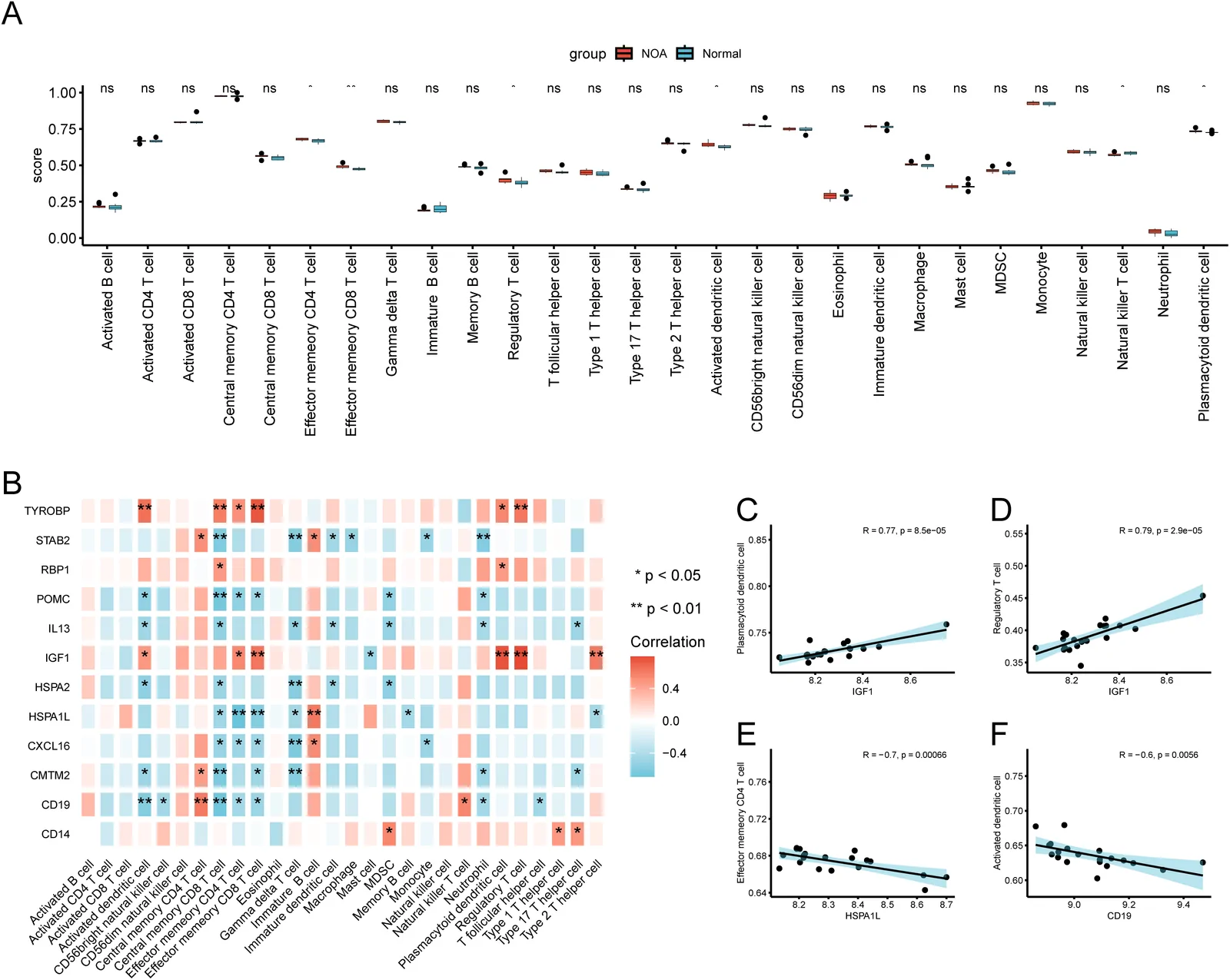
Immune microenvironment remodeling in NOA patients. (A) ssGSEA enrichment scores for 28 immune cell types in NOA vs OA control samples. Significant differences were assessed using Wilcoxon rank-sum test (*P* < 0.05). Key altered populations include effector memory CD4 ⁺ /CD8 ⁺ T cells, regulatory T cells, activated dendritic cells, natural killer T cells, and plasmacytoid dendritic cells. (B) Heatmap showing Spearman correlation coefficients between *Hub NOA.Sig* genes and immune cell infiltration levels. (C–F) Scatter plots illustrating significant correlations: (C) *IGF1* vs plasmacytoid dendritic cells (r = 0.765, *P* = 8.52e^−5^), (D) *IGF1* vs Tregs (r = 0.794, *P* = 2.87e^−5^), (E) *HSPA1L* vs effector memory CD4 ⁺ T cells (r = −0.695, *P* = 6.65e^−4^), (F) *CD19* vs activated dendritic cells (r = −0.595, *P* = 5.61e^−3^).

### 3.6. Computational screening prioritizes candidate small molecules for further investigation

To explore potential pharmacological modulators of the Hub NOA.Sig genes, we performed an integrative computational screening using the Drug–Gene Interaction Database (DGIdb; https://dgidb.org/) and the Connectivity Map (CMap; https://clue.io/). As illustrated in the drug–gene interaction network (Fig 6A), multiple small molecules were computationally associated with individual Hub NOA.Sig genes (green circles), represented as yellow squares.

**Fig 6:**
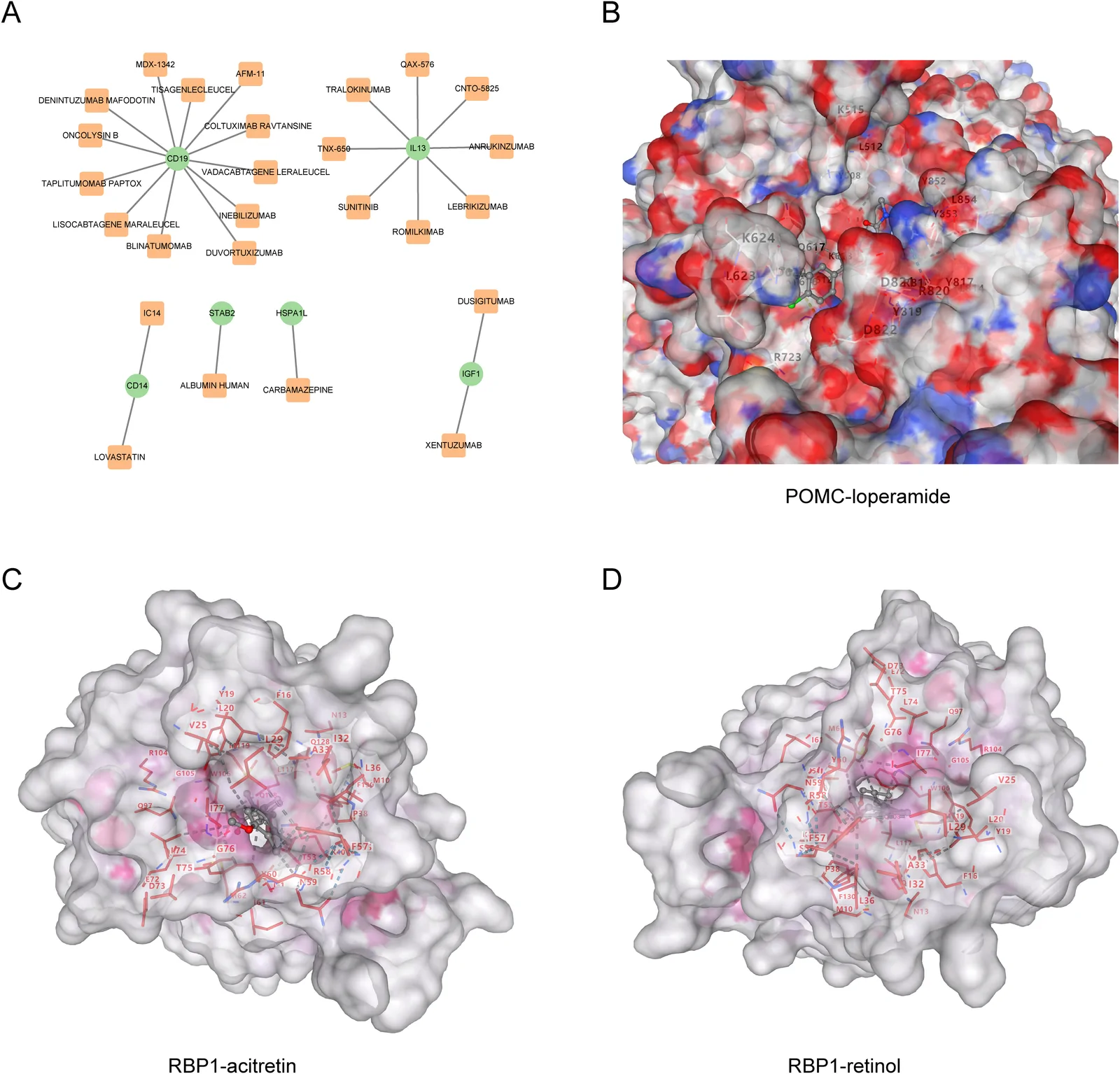
Computational drug–gene interactions and molecular docking predictions. Network of *Hub NOA.Sig* genes (green) and associated small molecules (yellow) from DGIdb/CMap. (B–D) Predicted binding poses of loperamide–POMC, acitretin–RBP1, and retinol–RBP1, respectively.

Subsequent analysis leveraging CMap connectivity scores prioritized three high-confidence drug–target pairs: *loperamide–POMC*, *acitretin–RBP1*, and *retinol–RBP1*. To assess the structural plausibility of these interactions, we conducted molecular docking simulations using CB-Dock2 (https://cadd.labshare.cn/cb-dock2/php/index.php). The resulting models suggest potential binding modes and favorable affinities for loperamide with POMC (Fig 6B), acitretin with RBP1 (Fig 6C), and retinol with RBP1 (Fig 6D). These findings highlight candidate compounds that warrant further experimental validation to evaluate their biological relevance in the context of NOA.

## 4. Discussion

The pathogenesis of NOA is closely accompanied by disordered testicular immune microenvironment homeostasis, and accumulating evidence indicates that immune-related molecular alterations are potentially involved in the progression of NOA [6]. Current therapeutic options for NOA remain limited and mainly rely on microsurgical sperm retrieval combined with assisted reproductive technology, which often yields unsatisfactory success rates and is inapplicable to patients with complete spermatogenic failure. Moreover, few available interventions target the underlying molecular and immunological abnormalities potentially associated with NOA. This unmet clinical need highlights the necessity to characterize the landscape of the testicular immune microenvironment and its putative correlation with NOA pathogenesis, which may provide novel mechanistic clues and therapeutic clues for future research. In this study, we identified Hub NOA.Sig, a 12-gene immune-related signature (CD14, CD19, CMTM2, CXCL16, HSPA1L, HSPA2, IGF1, IL13, POMC, RBP1, STAB2, TYROBP). This signature correlates with distinct immune infiltration patterns and provides observational insights into NOA immunological characteristics based on multi-cohort integrative analysis. Collectively, the 12 hub genes within Hub NOA.Sig can be categorized into several functional modules, which jointly reflect disrupted testicular immune homeostasis underlying NOA.

Innate immunity and pattern recognition molecules include *CD14* and *TYROBP*. *CD14* serves as a core TLR4 co-receptor that mediates innate inflammatory sensing. Its dysregulated expression has been documented in previous NOA transcriptomic studies, implying enhanced local inflammatory activation within testicular tissues [13,26]. *TYROBP* acts as an essential adaptor in myeloid and antigen-presenting cell signaling, and its abnormal upregulation has been linked to perturbed immune cell activation in NOA [27].

Chemokine and Th2 signaling-related genes encompass *CXCL16* and *IL13*. As a multifunctional chemokine, *CXCL16* regulates lymphocyte recruitment and has been repeatedly reported to be abnormally expressed in NOA transcriptomic profiles [28,29]. *IL13* modulates macrophage polarization toward an M2-like phenotype and participates in tissue fibrotic remodeling; its dysregulation is consistent with prior evidence linking Th2 immune imbalance to impaired spermatogenic recovery [30].

Stress response and immune regulatory genes consist of *HSPA1L, HSPA2,* and *CMTM2*. As key members of the HSP70 family, *HSPA1L* and *HSPA2* are indispensable for germ cell protein folding and meiotic maturation, while also functioning as damage-associated molecular patterns under cellular stress. Their differential expression in NOA has been validated in previous transcript and protein profiling studies [31]. *CMTM2* acts as a modulator of immune checkpoint stability; although its reproductive function remains underexplored, its enrichment in this signature implies potential involvement in the breakdown of testicular immune tolerance [32].

Metabolic and neuroendocrine–immune crosstalk genes include *IGF1, RBP1,* and *POMC*. *IGF1* modulates Sertoli cell activity and macrophage polarization, and its expression disorder has been closely associated with spermatogenic impairment in prior NOA investigations [33,34]. *RBP1* participates in retinol transport and retinoic acid metabolism, which are critical for meiosis and immune cell differentiation, supporting its potential relevance to NOA-related metabolic and immune disturbance [35]. *POMC* is involved in local inflammatory regulation and represents a newly identified correlate in NOA, requiring further experimental validation of its mechanistic role [36].

Immune cell marker and homeostasis-related genes comprise *CD19* and *STAB2*. *CD19* is a canonical B-cell marker rarely detected in normal testicular tissues, and its aberrant correlation with NOA raises the possibility of atypical lymphocyte infiltration in pathological conditions [37]. *STAB2* is implicated in endothelial and immune homeostasis, whereas its specific function in NOA pathogenesis remains poorly characterized in existing literature.

Notably, most hub genes identified herein have been previously reported at the transcript or protein level in independent NOA cohorts, which supports the reliability of our signature. Several newly highlighted members also provide unexplored clues for understanding NOA immune heterogeneity. Grouping these genes by functional categories effectively streamlines interpretation, reduces scattered gene-by-gene description, and balances observational findings with published evidence, thus avoiding excessive speculative inference.

Our observational findings extend previous NOA studies that mainly focused on individual inflammatory cytokines (e.g., IL-6, TNF-α) or limited immune cell populations based on small sample cohorts [38,39]. By integrating WGCNA, differential expression analysis and consensus multi-algorithm feature selection (LASSO, Boruta, Bagged Trees), our analytical framework reduces overfitting risk and improves the robustness of biomarker screening.

Immune deconvolution analysis revealed apparent differences in the abundance of multiple immune cell subsets in NOA testicular tissues, including effector memory CD4 ⁺ /CD8 ⁺ T cells, regulatory T cells, activated dendritic cells and plasmacytoid dendritic cells. Correlation analyses showed that *CD14* and *TYROBP* expression levels are positively correlated with monocyte/macrophage abundance, while *CXCL16* is associated with NKT cell infiltration. The testis is an immune-privileged organ maintained by intact blood-testis barrier, immunosuppressive mediators (e.g., TGF-β) and specialized peritubular cells. The observed immune microenvironment alterations may reflect disrupted immune homeostasis, which could be correlated with damage-associated molecular patterns released by stressed germ cells or abnormal *TLR/CD14* signal activation. Furthermore, unsupervised clustering based on *Hub NOA.Sig* expression profiles enabled the stratification of NOA patients into two molecular subtypes. Cluster A presents elevated expression of *CD14, TYROBP, CXCL16* and *IL13,* accompanied by enrichment in NF-κB signaling, cytokine–cytokine receptor interaction and leukocyte transendothelial migration pathways, suggesting a tendency toward inflammatory microenvironment alteration. By comparison, Cluster B shows relatively lower immune gene activity but more obvious downregulation of spermatogenesis-related genes such as *HSPA2* and *SYCP3,* implying a potential tendency toward intrinsic germ cell developmental abnormality. Such molecular subgrouping indicates the heterogeneous characteristics of NOA pathogenesis: some cases may be correlated with inflammatory immune microenvironment alternation, while others are potentially associated with primary germ cell developmental arrest accompanied by secondary immune changes. Unlike conventional NOA classification relying merely on hormonal indicators or histopathological scoring (e.g., Johnsen score) [40], the molecular subtypes identified in this study provide observational clues reflecting underlying pathological characteristics (immune microenvironment dysregulation versus intrinsic spermatogenic abnormality), which may support subtype classification and individualized therapeutic exploration in future clinical practice. Such molecular subtyping may provide references for stratified clinical management: for instance, anti-inflammatory or immunomodulatory strategies might be considered for Cluster A patients, while hormonal regulation or stem cell-based intervention approaches could be applicable for Cluster B cases. Compared with traditional histopathological typing (such as Sertoli cell-only syndrome and maturation arrest) that does not involve molecular and immune characteristics, our data-driven subtype analysis integrates immune and cellular molecular profiles, providing a more refined reference framework for subsequent mechanism research and targeted therapeutic exploration.

Our integrative computational approach screened several candidate small molecules, including loperamide, acitretin, and retinol, which exhibited inverse expression correlation patterns with the *Hub NOA.Sig* signature. Molecular docking further predicted relatively favorable binding affinity of loperamide to *POMC*, as well as acitretin and retinol to *RBP1*, with predicted binding energies ranging from −7.6 to −8.2 kcal/mol. It should be clearly emphasized that all drug–gene associations and docking results in the present study are merely in silico computational predictions rather than validated therapeutic evidence. These compounds are preliminarily prioritized only as candidate molecules for subsequent mechanistic and pharmacological exploration, and no direct clinical therapeutic applicability is claimed in this work.

To provide a reasonable biological background for these computational findings, we further summarized the potential links between these compounds and testicular microenvironment regulation. Retinol and its metabolite retinoic acid are well-established regulators of spermatogenic cell differentiation, meiosis progression, and testicular immune homeostasis; altered retinoid metabolism is closely correlated with impaired spermatogenesis. Acitretin, as a retinoid derivative, participates in epithelial cell differentiation and inflammatory modulation, implying its potential indirect relevance to testicular immune microenvironment remodeling. Loperamide, although primarily known as an opioid receptor agonist, has also been reported to exert modulatory effects on inflammatory responses in multiple peripheral tissues, suggesting a plausible biological basis for its potential correlation with immune-related genes such as *POMC.* Collectively, these compounds may serve as promising exploratory candidates for future basic experimental validation, whereas their exact biological function, testicular permeability, blood–testis barrier penetration, and potential reproductive toxicity remain to be experimentally verified.

Several limitations should be acknowledged. First, our analyses rely on bulk RNA-seq data obtained from heterogeneous testicular tissues. NOA is characterized by extensive germ cell loss and profound alterations in testicular cellular composition, accompanied by somatic cell immaturity, aberrant growth factor signaling, and enhanced inflammatory responses [41]. These inherent cellular perturbations may introduce composition-driven confounding bias, such that the identified immune signatures and predicted immune infiltration patterns may reflect secondary shifts in cell population proportions rather than primary immune activation. Accumulated evidence further confirms that immature Sertoli cells are prevalent in atrophic seminiferous tubules across NOA subtypes, which further distorts tissue cellular constitution [42]. Such changes may overestimate or obscure the actual infiltration level of specific immune subsets. Therefore, we caution that the remodeling of the testicular immune microenvironment and correlations between hub genes and immune cells should be interpreted prudently, fully accounting for confounding effects derived from altered cellular proportions in bulk transcriptomic data. Single-cell or spatial transcriptomic analyses are required in future work to resolve cell-type-specific expression and validate independent immune regulatory mechanisms in NOA. Second, we acknowledge that obstructive azoospermia (OA) samples were adopted as control subjects. Given the limited accessibility of normal human testicular tissues from fertile individuals, OA samples represent the most ethically acceptable and publicly available alternative in current transcriptomic research. However, OA cannot fully recapitulate physiological testicular homeostasis and may introduce notable confounding effects, particularly in immune-related analyses. Although spermatogenic function is largely preserved in OA, chronic intraluminal pressure elevation and epididymal fluid accumulation can retrogradely disrupt the seminiferous tubular microenvironment and initiate low-grade aseptic inflammation. Previous studies have documented focal infiltration of macrophages and lymphocytes in OA testicular tissues, alongside elevated pro-inflammatory mediators (e.g., IL-6, TNF-α) and subtle disruption of blood-testis barrier integrity. Consequently, the differential immune profiles identified between NOA and OA may not exclusively reflect disease-intrinsic immune activation in NOA, but may also partially capture secondary inflammatory perturbations inherent to OA pathology. This inherent bias risks overestimating the infiltration abundance of effector memory T cells and regulatory T cells, or misinterpreting OA-related basal inflammation as NOA-specific immune signatures. The expression patterns of hub genes such as *CD14* and *CXCL16* may likewise be diluted or masked by baseline immune alterations within the OA control group. Future external validation utilizing strictly defined normal testicular specimens from fertile donors will help eliminate such confounding artifacts and better delineate NOA-specific immune molecular patterns. Third, it should be clearly emphasized that all findings in this study are derived from bulk transcriptomic data, which can only support the analysis of correlative association rather than direct causal inference. Although the identified gene signature and enrichment results support the involvement of immune molecular characteristics in NOA, we avoid overinterpreting these observational correlations as definite causal relationships or direct immune-mediated tissue damage. All findings in this study are framed as hypothesis-generating observational results and require further experimental validation. These correlative molecular signatures and immune alteration patterns only provide candidate targets and potential research directions for subsequent mechanism exploration, rather than confirming definite pathogenic mechanisms. Fourth, incomplete clinical metadata of the included datasets limited our ability to further analyze the correlation between molecular subtypes and clinical endpoints, such as TESE success rate and detailed histopathological classification (e.g., Sertoli cell-only syndrome versus maturation arrest).This study did not adopt a nested cross‑validation framework to separate feature selection from model evaluation. In addition, the initial DEG screening integrated all datasets, including those later used as independent validation cohorts such as GSE45885. This may introduce information leakage and optimistic bias, potentially overestimating the model’s diagnostic performance and generalization ability across external cohorts. Therefore, the diagnostic efficacy of the current signature should be interpreted with caution, and further validation in fully independent prospective cohorts is warranted in future studies.

We acknowledge that reporting AUC confidence intervals or standard deviations is valuable for evaluating model stability. However, the training cohort has a limited sample size, and each fold in cross‑validation contained a small number of samples. Moreover, feature selection was not independently re‑executed within each fold. Directly calculating AUC variability under such conditions would mainly reflect random fluctuations caused by small sample partitioning, rather than true generalization error on unseen data. Forcing such statistics may mislead readers into regarding them as external validation stability. Accordingly, we employed cross‑cohort consistency across multiple independent GEO datasets to reflect model robustness, and explicitly explained this methodological consideration here. Future studies with larger sample sizes or nested resampling designs will enable more reliable estimation of model performance variability. Fifth, the core training dataset GSE145467 had a limited sample size of only 20 cases. Small sample training inevitably increases the risk of overfitting when multiple machine learning algorithms are applied, and the internal cross-validation performance may therefore be subject to subtle optimistic bias. Sixth, all transcriptomic data were retrieved from multiple GEO datasets generated on different microarray platforms. No formal batch correction or cross-platform normalization was performed in the present work. Instead, we adopted a consensus filtering strategy by retaining genes consistently differentially expressed across at least two datasets to mitigate batch-related bias. Nevertheless, this approach cannot completely eliminate latent batch effects, and some genuine biological signals masked by technical heterogeneity may be missed. Future studies should integrate standardized batch correction procedures and independent experimental validation to verify and refine the present signature.

Nevertheless, *Hub NOA.Sig* provides a robust immune-related molecular framework for understanding the heterogeneous molecular characteristics of NOA. This signature enables accurate transcriptomic classification, supports molecular subtyping, and offers candidate genes and small molecule compounds for subsequent mechanism exploration and pharmacological research. Testicular biopsy remains an invasive clinical examination; future research can further explore whether secretory hub genes such as *CXCL16* and *IL13* can be detected in seminal plasma or peripheral blood, which may facilitate the development of non-invasive molecular typing methods. Such integrative bioinformatics analysis may complement conventional research frameworks and provide candidate molecular clues for future NOA mechanistic studies.

Future research is warranted to validate the diagnostic stability and clinical correlation of *Hub NOA.Sig* in prospective independent cohorts, integrate multi-omics data including proteomics and epigenomics, and perform functional verification of core hub genes and candidate compounds via cellular and animal models. Bridging molecular observational discovery with clinical translational exploration will help promote the precise diagnosis and comprehensive management of NOA.

In summary, based on multi-cohort transcriptomic data combined with WGCNA and consensus machine learning algorithms, we identified a robust 12-gene immune-related signature correlated with NOA. This signature reflects heterogeneous immune-related molecular features and provides candidate molecules for subsequent experimental exploration. Although further experimental and clinical validation is still required, our observational findings provide a valuable transcriptomic reference framework for understanding the molecular and immune characteristics of NOA.

## Supporting information

S1 Table Immune-related genes obtained from the ImmPort database. (XLSX)

S2 Table Differential expression analysis of GSE145467 (NOA vs OA). (XLSX)

S3 Table Differential expression analysis of GSE45885 (NOA vs OA). (XLSX)

S4 Table Differential expression analysis of GSE45887 (NOA vs OA). (XLSX)

S5 Table Differential expression analysis of GSE9210 (NOA vs OA). (XLSX)

S6 Table Diagnostic performance of five machine learning classifiers after 50 repeated five-fold cross-validation on the training cohort (GSE145467). (XLSX)
