# Supplementary material for: Integrated bioinformatics analysis identifies an immune-related gene signature associated with non-obstructive azoospermia
Source: PLoS One. 2026 Oct 12;21(10):e0360305. doi: 10.1371/journal.pone.0360305 (PMC13657319; doi:10.1371/journal.pone.0360305)
Supplement: Supplementary file 1 [file pone.0360305.s007.pdf]

Reviewer Comments on Manuscript

Title: *Integrated Bioinformatics Analysis Identifies an Immune-Related Gene Signature Associated with Non-Obstructive Azoospermia*

A. Overall Assessment

This manuscript presents an integrative bioinformatics analysis aimed at identifying immune-related biomarkers associated with non-obstructive azoospermia (NOA). The authors analyze four publicly available GEO datasets and combine differential expression analysis, WGCNA, and multiple machine-learning algorithms to derive a 12-gene immune signature (Hub NOA.Sig). The model demonstrates reasonable diagnostic performance (AUC = 0.87) and identifies potential immune infiltration patterns and candidate drug-gene interactions.

The study addresses an important problem in reproductive medicine, and the use of multiple datasets and computational approaches is commendable. However, several methodological issues, clarity problems, and limitations regarding validation and interpretation reduce the overall impact of the work. Substantial clarification and methodological rigor are needed before the manuscript can be considered for publication.

B. Major Comments

1. Lack of Experimental and Biological Validation

The study relies entirely on **in-silico analysis** using publicly available datasets.

- **No Protein-Level Confirmation**: Although the 12-gene "Hub NOA.Sig" was identified across multiple transcriptomic datasets, there is no validation using techniques like immunohistochemistry (IHC) or Western blotting to confirm that these genes are actually translated into proteins within the testicular tissue.
- **No Functional Assays**: The study lacks *in vitro* or *in vivo* experiments to demonstrate that the identified genes (e.g., *CMTM2* or *STAB2*) directly influence spermatogenesis or immune cell recruitment.
- **Transcriptional vs. Translational Gap**: Gene expression levels do not always correlate with protein abundance or activity, especially in the testis where complex post-translational regulation occurs during meiosis and spermiogenesis.

2. Resolution and Cellular Specificity Issues

Because the study relies on **bulk RNA sequencing** data, it cannot definitively identify the cellular source of the gene signature.

- **Cellular Origin Ambiguity**: It remains unclear whether the "immune" signature originates from infiltrating immune cells (like T cells or macrophages) or from resident testicular cells (Sertoli or Leydig cells) adopting an inflammatory phenotype.
- **Absence of Single-Cell Data**: The manuscript does not integrate single-cell RNA sequencing (scRNA-seq), which is now the gold standard for dissecting the specific cellular microenvironment of the testis. This prevents a clear understanding of how these 12 genes interact across different cell types.

3. Dataset heterogeneity and batch effects

The four GEO datasets originate from **different platforms (GPL4133, GPL6244, GPL887)** and likely contain significant batch effects. However, the manuscript does not clearly describe:

- Whether **batch correction** was performed
- How expression values were normalized across datasets
- Whether cross-platform integration methods (e.g., ComBat or similar approaches) were used

Without proper batch correction, the differential expression analysis and machine learning results may be confounded.

4. Sample size limitations for model training

The training dataset (GSE145467) includes only **20 samples (10 NOA and 10 OA)**. Training multiple machine learning models on such a small dataset raises concerns about **model stability and overfitting**, even with cross-validation.

The authors should provide:

- Justification for using this dataset as the primary training set
- Additional validation metrics
- Confidence intervals for AUC values

5. Machine learning methodology needs clarification

The manuscript uses multiple algorithms (LASSO, Boruta, LVQ, Bayesian, Treebag), but the **feature selection process is not fully transparent**.

Important details are missing:

- Hyperparameter settings

- Feature selection thresholds
- Whether nested cross-validation was used
- How model performance variability was assessed

These details are necessary for reproducibility.

6. Biological interpretation of hub genes remains speculative

While the discussion provides literature-based explanations for individual genes, the **functional link between these genes and NOA pathogenesis remains largely speculative**.

More focused pathway analysis or functional enrichment (e.g., GO/KEGG analysis) would help clarify the biological relevance of the identified signature.

7. Drug-gene interaction and molecular docking results are preliminary

The identification of potential compounds (loperamide, acitretin, retinol) is interesting but appears **highly exploratory**. The biological relevance of these predicted interactions with **POMC and RBP1** in the context of testicular physiology is unclear.

The authors should moderate their claims and clearly state that these results represent **hypothesis-generating predictions** rather than therapeutic recommendations.

8. Missing Contextual Information

- **Etiological Heterogeneity**: NOA can be caused by various factors, including genetic deletions (AZF), Klinefelter syndrome, or cryptorchidism. The manuscript does not sub-analyze whether the 12-gene signature performs differently across these distinct clinical etiologies.
- **CD19 Mystery**: The inclusion of **CD19** (a B-cell marker) is unusual since B-cells are rarely reported in healthy or diseased human testes. The lack of a biological explanation or confirmatory staining for this finding is a notable gap.

C. Minor Comments

1. Terminology inconsistency

The manuscript inconsistently uses the terms:

- *Hub NOA.Sig*
- *Hub NOA.sig*
- *NOA.sig*

These should be standardized throughout the manuscript.

2. Clarity in Methods

Some methodological descriptions are vague. For example:

- Section 2.4 lists **six machine learning algorithms**, but only five are described earlier.
- The description of **Bayesian algorithm** is unclear.

More detailed methodological explanations are needed.

3. Statistical thresholds

The manuscript states that **$P \leq 0.05$** was used to define differential expression. However, **multiple testing correction (e.g., FDR adjustment)** is not described.

Given the large number of genes analyzed, correction for multiple testing is necessary.

4. Figures require better explanation

Several figures (e.g., Figures 3–6) lack sufficient explanation in the text. The figure legends should clearly describe:

- Data sources
- Statistical tests
- Interpretation of visual elements

D. Recommendation

Major Revision

The study presents an interesting computational approach to identifying immune-related signatures associated with non-obstructive azoospermia. However, methodological clarification, stronger validation, and more cautious interpretation of results are necessary before the manuscript can be considered for publication.
