# Supplementary material for: Integrated bioinformatics analysis identifies an immune-related gene signature associated with non-obstructive azoospermia
Source: PLoS One. 2026 Oct 12;21(10):e0360305. doi: 10.1371/journal.pone.0360305 (PMC13657319; doi:10.1371/journal.pone.0360305)
Supplement: Supplementary file 2 [file pone.0360305.s010.pdf]

Reviewer #2 (Second Revision)

I appreciate the authors' careful and thorough revision of the manuscript. The responses provided in the rebuttal letter are clear and comprehensive, and the corresponding changes have been appropriately incorporated into the revised manuscript. My previous concerns have been satisfactorily addressed, as summarized below.

1. WGCNA Minimum Module Size Inconsistency

Previous comment: The manuscript contained an inconsistency regarding the minimum module size used for WGCNA, reporting both 50 and 10 in different sections.

Assessment: The revised manuscript now consistently reports a minimum module size of **50** throughout both the Methods (Section 2.3) and Results (Section 3.2). The inconsistency has been fully resolved.

---

2. Control Group Terminology

Previous comment: References to "normal testis" or "normal controls" should be replaced with "OA controls" throughout the manuscript, figures, and tables to accurately reflect the comparator group.

Assessment: The manuscript now consistently refers to the comparator group as **OA controls** throughout the Methods, Results, and Discussion. The remaining reference to "normal human testicular tissues" in the Limitations section is appropriate because it specifically refers to truly fertile donor tissue that was unavailable for this study and is clearly distinguished from the OA controls used in the analysis. The authors also indicated that Figure 2H and Figure 5A were updated accordingly; these figure labels should simply be verified in the final production files. Overall, this concern has been satisfactorily addressed.

---

3. Translational Language in the Abstract

Previous comment: The Abstract contained language that overstated the translational implications of the study and should better reflect the exploratory nature of the findings.

Assessment: The authors have appropriately revised the Abstract. The study objective is now presented in a more exploratory manner, the description of the gene signature has been moderated, "diagnostic performance" has been replaced with "classification performance," and the discussion of drug repurposing has been revised to emphasize candidate drug-gene pairs requiring future experimental validation. The concluding statement has likewise been

appropriately tempered. These revisions substantially improve the balance and scientific accuracy of the Abstract.

---

4. Machine Learning Evaluation

Previous comment: Confidence intervals or standard deviations for the reported AUC values should be provided to better characterize model performance during repeated cross-validation.

Assessment: The revised manuscript now reports results from **50 rounds of repeated five-fold cross-validation**, including mean AUC, standard deviation, and 95% confidence intervals for all five machine learning classifiers in Supplementary Table S6. The accompanying explanation appropriately notes that these statistics reflect variability from repeated resampling rather than external validation, which is scientifically appropriate. This concern has been fully addressed.

Minor Editorial Observation

I noted one minor wording inconsistency in Section 3.3, where the manuscript states that **"six machine learning algorithms"** were evaluated, although only **five** algorithms are listed (Bagged Trees, Bayesian, Boruta, LASSO, and LVQ), consistent with the Methods, Abstract, and Figure 3 legend. This appears to be a typographical error and can be corrected during copyediting.

Overall Recommendation

The authors have addressed all of my previous comments thoroughly and appropriately. The revisions have improved the methodological clarity, terminology, and presentation of the manuscript, and I have no further scientific concerns. I recommend **acceptance** after correction of the minor editorial issue noted above.
