# Supplementary material for: Integrated bioinformatics analysis identifies an immune-related gene signature associated with non-obstructive azoospermia
Source: PLoS One. 2026 Oct 12;21(10):e0360305. doi: 10.1371/journal.pone.0360305 (PMC13657319; doi:10.1371/journal.pone.0360305)
Supplement: Supplementary file 3 [file pone.0360305.s008.docx]

Dear Editor,

Your comments, along with those of the other reviewers, are extremely important to our article. We greatly appreciate your insights, which have significantly contributed to enhancing the quality of our work. In this revision, we have prepared a detailed point-by-point response letter for you and the other valued reviewers. This letter acknowledges your feedback and clearly outlines the revisions we have made.

In response to the reviewers' comments, we have made extensive revisions to the manuscript. If there are any further modifications you would like us to consider, we would be more than willing to accommodate them, and we genuinely appreciate your assistance. *PLOS ONE* is a highly esteemed journal, and we hope that our manuscript can be considered for publication. Thank you very much for your support.

We would like to express our sincere gratitude to you and the reviewers for your valuable comments on our manuscript. In light of the thorough and insightful feedback provided, we have revised the manuscript accordingly. We have added relevant content to address the reviewers' suggestions, and we have rewritten several sections to ensure clarity and completeness. We hope that these modifications meet the reviewers' expectations and enhance the overall quality of the manuscript.

Thank you again sincerely for your time, effort, and kind support.

Best regards,

All authors

Reviewers' comments:

Reviewer's Responses to Questions

**Comments to the Author**

1. Is the manuscript technically sound, and do the data support the conclusions?

The manuscript must describe a technically sound piece of scientific research with data that supports the conclusions. Experiments must have been conducted rigorously, with appropriate controls, replication, and sample sizes. The conclusions must be drawn appropriately based on the data presented.

Reviewer #1: Partly

Reviewer #2: Partly

2. Has the statistical analysis been performed appropriately and rigorously?

Reviewer #1: Yes

Reviewer #2: No

3. Have the authors made all data underlying the findings in their manuscript fully available?

The [PLOS Data policy](http://track.editorialmanager.com/CL0/http://www.plosone.org/static/policies.action%23sharing/1/010f019d84d65956-7e02b41a-c754-40a7-97cc-d44275ad9caf-000000/VTsSdKSKE5Ek529MgowMDbIF-6IyQz9-VpJCL8bKQB4=258) requires authors to make all data underlying the findings described in their manuscript fully available without restriction, with rare exception (please refer to the Data Availability Statement in the manuscript PDF file). The data should be provided as part of the manuscript or its supporting information, or deposited to a public repository. For example, in addition to summary statistics, the data points behind means, medians and variance measures should be available. If there are restrictions on publicly sharing data—e.g. participant privacy or use of data from a third party—those must be specified.

Reviewer #1: Yes

Reviewer #2: No

4. Is the manuscript presented in an intelligible fashion and written in standard English?

PLOS ONE does not copyedit accepted manuscripts, so the language in submitted articles must be clear, correct, and unambiguous. Any typographical or grammatical errors should be corrected at revision, so please note any specific errors here.

Reviewer #1: Yes

Reviewer #2: No

5. Review Comments to the Author

Please use the space provided to explain your answers to the questions above. You may also include additional comments for the author, including concerns about dual publication, research ethics, or publication ethics. (Please upload your review as an attachment if it exceeds 20,000 characters)

Reviewer #1: Reviewer Comments

Summary
This study investigates immune-related biomarkers in non-obstructive azoospermia (NOA) through the analysis of four independent transcriptomic datasets. The authors identify a 12-gene immune-associated signature (Hub NOA. Sig) using an integrative pipeline that combines differential expression analysis, weighted gene co-expression network analysis (WGCNA), and multiple machine learning approaches. The diagnostic performance of this signature is validated across independent cohorts. The study further explores altered immune cell populations, proposes two molecular subtypes, and identifies potential druggable targets through drug–gene interaction analysis and molecular docking.

The manuscript is generally well structured, and the use of multiple datasets along with an integrative analytical approach is a clear strength. The topic is relevant, and the work provides a useful resource for understanding NOA at the transcriptomic level. However, several aspects of the biological interpretation and presentation would benefit from clarification and more cautious framing.

Comments
1. A key limitation of the study is the use of bulk RNA-seq data from heterogeneous testicular tissue. As NOA is associated with significant changes in cellular composition (e.g., germ cell loss), the observed immune signatures may reflect shifts in cell populations or secondary effects rather than primary immune activation.
Suggestion: Although this is mentioned briefly in the discussion, it would strengthen the manuscript to emphasize this limitation more clearly and elaborate on how it may influence the interpretation of immune infiltration results.
RE: Thank you very much for pointing out this key limitation. We fully agree that the bulk RNA-seq data derived from heterogeneous testicular tissues used in our study introduces cellular composition bias, which is mainly attributed to the massive loss of spermatogenic cells in NOA patients. Accordingly, the identified immune signatures may partially reflect relative proportional changes of cell populations or secondary biological effects, instead of bona fide primary immune activation. This issue directly undermines the reliability of immune infiltration abundance estimation and the subsequent biological interpretation.

In the revised manuscript, we will significantly strengthen the discussion of this limitation. We will elaborate in detail how alterations in cellular composition may overestimate or confound the infiltration levels of specific immune cell subsets. Furthermore, we will remind readers to cautiously interpret the remodeling of the testicular immune microenvironment and the correlations between genes and immune cells, and to fully recognize such confounding effects driven by shifts in cellular proportions. This revision will greatly improve the academic rigor of our study and the robustness of our conclusions.

For the specific details of the changes, please refer to the first item under “Limitations” in the Discussion section; I have marked the revised parts in red font.

Thank you again for your valuable comments and constructive suggestions.

2. The use of obstructive azoospermia (OA) samples as controls is understandable given the limited availability of datasets on normal testicular tissue. However, OA does not fully represent normal physiology and may introduce confounding, particularly in immune-related analyses.
Suggestion: Please expand the discussion on how OA differs from normal testicular conditions and how this might affect the interpretation of the results.
RE:Thank you very much for your careful and constructive comments. We fully agree with this valuable suggestion and have substantially expanded the discussion regarding the potential confounding effects of using obstructive azoospermia (OA) samples as controls in the revised manuscript.

Although employing OA samples as controls represents the most feasible alternative under current public dataset limitations, their potential interference with immune-related interpretations should be interpreted with caution. OA is primarily caused by mechanical obstruction of the reproductive tract, with largely preserved intrinsic spermatogenic function. Nevertheless, long-term elevation of intraluminal pressure and epididymal fluid stasis can retrogradely alter the microenvironment of seminiferous tubules and trigger low-grade aseptic inflammation. Accumulated evidence has confirmed increased focal infiltration of macrophages and lymphocytes in OA testicular tissues, accompanied by upregulated pro-inflammatory cytokines such as IL-6 and TNF-α in epididymal fluid, as well as mild rearrangement of tight junction proteins within the blood-testis barrier.

Accordingly, the observed immune differences between the NOA and OA groups may not entirely reflect primary immune activation inherent to NOA pathogenesis but may partially represent secondary immune perturbations under OA background conditions. Such bias may overestimate the infiltration abundance of immune subsets such as effector memory T cells and regulatory T cells, or misclassify low-grade inflammatory characteristics concomitant with OA as specific immune signatures of NOA. In our analyses, the differential expression of hub genes including CD14 and CXCL16 may genuinely reflect immune microenvironment remodeling in NOA, while also being partially diluted or masked by relative immune activation in the OA control group.

In future studies, incorporating strictly screened testicular tissues from fertile males (e.g., organ donors or individuals undergoing gender reassignment surgery) as external validation controls would help decouple disease-specific immune signatures and further strengthen the objectivity of our conclusions.

We have supplemented detailed descriptions in the Limitations section of the revised manuscript to elaborate that, despite mechanical obstruction as the primary cause of OA, persistent intraductal pressure elevation and microenvironmental disturbance may induce secondary inflammatory responses and immune cell infiltration, substantially deviating the testicular immune landscape from physiological normal status. Supported by existing literature, we further interpret how such deviations may introduce false-positive immune activation signals or overestimate the infiltration levels of effector memory T cells and other immune subsets. This supplementation will guide readers to prudently interpret the observed immune subset alterations. We also acknowledge the necessity of recruiting normal fertile testicular controls for external validation in future work, thereby substantially improving the objectivity and rigor of result interpretation without additional experimental workload.

Thank you again for your insightful comments.

3. The manuscript places strong emphasis on immune dysregulation as a driver of NOA. While the identified genes and enrichment analyses support immune involvement, the conclusions at times suggest causality.
Suggestion: Given that the study is based on bulk transcriptomic data, it would be helpful to more clearly distinguish between observed associations and proposed mechanisms. The authors should avoid language suggesting direct immune-mediated damage unless experimentally supported. Framing these findings as hypothesis-generating would improve clarity.
RE: Thank you very much for your careful and insightful comment. We fully recognize that bulk transcriptomic analysis alone cannot establish causal relationships. We acknowledge that certain statements in the original manuscript may have overinterpreted the results and implicitly suggested a causal role of immune dysregulation in driving NOA pathogenesis.

In the revised manuscript, we have systematically reviewed and revised the entire text. All conclusive and causal claims have been moderated into more conservative association-based descriptions. We have explicitly clarified that the Hub NOA.Sig and related immune-related alterations observed in this study should be regarded as hypothesis-generating findings that require further experimental validation. We have also emphasized that these correlative results merely provide candidate molecular targets and potential research directions for subsequent mechanistic exploration, rather than demonstrating direct immune-mediated pathogenic damage. This revision greatly improves the precision, clarity and scientific rigor of our conclusions.

Thank you again for your valuable suggestion.

4. The drug-gene interaction and molecular docking analysis is exploratory.
Suggestion: The authors should clearly state that these findings are based on computational predictions. Statements suggesting therapeutic applicability should be toned down, and, if possible, a brief biological rationale linking the identified compounds to NOA could be included.
RE: Thank you very much for your thoughtful comment. We fully agree that our drug–gene interaction screening and molecular docking analyses are essentially exploratory computational predictions, and we acknowledge that some descriptions implying direct therapeutic applicability should be appropriately moderated.

In the revised manuscript, we have thoroughly revised this section. We have explicitly clarified that all relevant results are purely computational predictions derived from bioinformatics analysis. We have deliberately toned down overstated claims and framed these compounds only as preliminary candidates obtained from in silico screening, while removing any wording that suggests immediate clinical or therapeutic potential. Furthermore, we have supplemented the Discussion with a brief biological rationale linking loperamide, acitretin and retinol to testicular physiology, immune regulation and spermatogenesis homeostasis, to better contextualize these computational predictions. These revisions make the exploratory findings more objective, cautious, and scientifically rigorous.

Thank you again for your valuable suggestion.

5. The identification of two molecular subtypes is interesting. However, the manuscript extends this finding to potential applications in personalized treatment.
Suggestion: In the absence of correlations with clinical outcomes, histopathology, or hormonal data, it would be more appropriate to present these subtypes as preliminary transcriptomic classifications. The discussion of clinical applicability should be moderated accordingly.
RE:Thank you very much for your careful and insightful review. We fully agree that it is premature to directly extend the identified two molecular subtypes to personalized treatment applications without valid correlations with clinical outcomes, histopathological features, and hormonal profiles.

In the revised manuscript, we have rigorously adjusted the relevant descriptions. We now explicitly define these two subtypes as preliminary transcriptomic classifications. We have appropriately moderated any overinterpretation regarding clinical applicability and therapeutic guiding value, and refrain from making direct clinical inferences. Meanwhile, we clarify that these molecular subtypes merely serve as a hypothesis-generating framework to provide exploratory directions for future studies integrating clinical phenotypic data. Such revisions ensure that our conclusions are fully consistent with the available evidence base.

Thank you again for your valuable and constructive comment.

6. The discussion is quite dense, particularly with gene-by-gene descriptions, and at times leans heavily toward speculative interpretation.
Suggestion: Grouping genes into functional categories may improve readability. It would also be useful to include a brief discussion on whether these immune signatures have been previously reported at the transcript or protein level in NOA studies.

RE:Thank you very much for your careful and valuable comments. We fully agree that the current Discussion section is relatively dense with gene-by-gene descriptions and occasionally contains overly speculative interpretations.

In the revised manuscript, we have reorganized these 12 hub genes into several functional categories, such as innate immunity and pattern recognition, chemokine and Th2 signaling, as well as stress and metabolic immune regulation. This grouping greatly improves the logical structure and readability of the discussion. In addition, we have supplemented each functional module with existing evidence from previous NOA studies, summarizing relevant transcript-level and protein-level reports of these immune signatures in the literature. This arrangement balances speculative interpretation with published experimental evidence, making the Discussion more concise, objective, and evidence-based.

Thank you again for your constructive suggestion.

Minor comments:
1. Repetition regarding model robustness and multi-cohort validation could be reduced for better readability.
2. The study workflow should be briefly described in the Results section (as is done in the figure legends). Abbreviations currently listed in the text may be moved to the legend of Figure 1.
3. In Figure 2, the text states that 1,793 genes exhibited differential expression in at least two datasets, whereas Figure 2E shows 1,749. Please ensure consistency.
RE:Thank you very much for your careful comments and valuable suggestions.

1. We have carefully checked the full manuscript and appropriately reduced repetitive descriptions regarding model robustness and multi-cohort validation. We only retain key information at essential positions in the Results and Discussion sections to improve overall readability and avoid redundant narration.

2. We have followed your suggestion and briefly summarized the overall study workflow at the beginning of the Results section. Meanwhile, all abbreviations originally explained in the main text have been moved to the legend of Figure 1 to simplify the body text layout and standardize manuscript writing.

3. We have carefully checked the numerical inconsistency between the main text and Figure 2E. The correct number has been unified and revised to ensure complete consistency between textual description and graphical presentation.

Thank you again for your thoughtful comments, which greatly help improve the clarity and standardization of our manuscript.

Recommendation:
Minor revision
The study is methodologically sound and suitable for publication after revisions that improve clarity, moderate interpretation, avoid overstating translational implications, and better align conclusions with the data.

Reviewer #2: Review Comments to the Author

The manuscript presents an integrative bioinformatics analysis to identify immune-related biomarkers associated with non-obstructive azoospermia (NOA). The authors analyze multiple publicly available datasets and apply differential expression analysis, weighted gene co-expression network analysis (WGCNA), and several machine learning approaches to identify a 12-gene immune-related signature associated with NOA. The topic is relevant, and the use of multiple datasets and computational strategies is commendable. However, several methodological and interpretational issues should be addressed to improve the rigor and clarity of the study.

Major Comments

Lack of experimental validation
The study relies entirely on computational analyses of public datasets. While such analyses are valuable for hypothesis generation, the absence of experimental validation limits the strength of the conclusions. Validation of key genes using independent experimental methods (e.g., qPCR, protein expression analysis, or validation in an independent clinical cohort) would significantly strengthen the manuscript.

RE:Thank you very much for your valuable comment. We fully acknowledge that this study is entirely based on computational analysis of public datasets, and the lack of independent experimental validation inevitably limits the robustness of our conclusions.

In the revised manuscript, we have explicitly emphasized this limitation in the Discussion section. We clearly state that the present work is hypothesis-generating exploratory research, and highlight that the identified Hub NOA.Sig genes, altered immune cell profiles, and molecular subtype classification all require further experimental verification, such as qPCR detection, protein expression analysis, or validation in an independent clinical cohort, before being regarded as definitive conclusions. We also specify concrete directions for subsequent experimental validation for each core finding, to properly interpret the reliability of current results and guide future study design.

We have already supplemented relevant statements in the Discussion section according to your suggestion in the revised version.

Thank you again for your constructive and insightful comment.

Machine learning methodology requires clarification
The manuscript employs multiple machine learning algorithms (LASSO, Boruta, LVQ, Bayesian, and Treebag) to identify hub genes; however, the methodological details are insufficient for reproducibility. Important aspects such as hyperparameter settings, feature selection criteria, and details of model optimization are not clearly described. In addition, it is unclear whether nested cross-validation was used to separate feature selection from model evaluation. The authors should provide more detailed methodological descriptions and report variability in model performance (e.g., confidence intervals or standard deviations of AUC values).

RE:Thank you very much for your careful and constructive comments. We fully agree that the machine learning methodology needs further clarification to improve methodological transparency and reproducibility.

First, we have supplemented detailed methodological descriptions in the Materials and Methods section regarding hyperparameter settings, feature selection criteria, and model optimization procedures for all five algorithms (LASSO, Boruta, LVQ, Bayesian, and Treebag). We have specified the R packages, iteration settings, cross‑validation strategies, key hyperparameters, and gene screening thresholds used in each algorithm, making the entire feature selection workflow fully reproducible.

Second, we acknowledge that nested cross‑validation was not adopted to separate feature selection from model evaluation in the current study. We have frankly stated this methodological limitation in the Discussion section. We clarified that this arrangement may introduce information leakage and optimistic bias, which could overestimate model generalization performance on external validation cohorts. Readers are reminded to interpret the diagnostic efficacy cautiously, and further validation in completely independent clinical cohorts is recommended in future work.

Third, we fully understand the importance of reporting performance variability such as AUC confidence intervals or standard deviations. Nevertheless, limited sample size in the training set, small fold‑wise validation subsets, and non‑nested feature selection make it inappropriate to directly calculate and report AUC variability metrics, which may produce misleading estimates rather than reflecting true generalization error. Therefore, we have explained this methodological consideration explicitly in the Discussion. Instead of forcing unreliable variability statistics, we use consistent performance across multiple independent GEO cohorts to reflect model robustness, and note that future larger‑sample or nested resampling designs are required for reliable estimation of performance variability.

We have completed all the above revisions and additions in the revised manuscript as suggested. Thank you again for your valuable comments, which greatly improve the methodological rigor and interpretability of our work.

Small sample size for model training
The primary training dataset contains a limited number of samples, which may increase the risk of overfitting when applying multiple machine learning algorithms. The authors should discuss this limitation more explicitly and provide additional justification for the robustness of the predictive model.

RE:Thank you very much for pointing out this limitation. We acknowledge that the limited sample size of the primary training dataset may increase the risk of overfitting when using multiple machine learning algorithms. We have added a concise explanation in the Discussion to explicitly state this concern, and meanwhile summarized multi-cohort validation evidence to justify the robustness of the model.

Thank you again for your constructive suggestion.

Dataset heterogeneity and batch effects
The study integrates multiple GEO datasets derived from different platforms. The manuscript does not clearly describe whether batch correction or cross-platform normalization methods were applied. Since batch effects can significantly influence differential expression and downstream analyses, clarification of the preprocessing and normalization steps is necessary.

RE:Thank you very much for raising this important methodological concern regarding dataset heterogeneity and batch effects. We have carefully checked our analytical pipeline and confirm that no dedicated batch correction or cross-platform normalization algorithms were applied in this study.

Instead, we adopted a consensus strategy: differential expression analysis was performed independently for each GEO dataset. We then integrated genes that were consistently differentially expressed in at least two datasets to filter robust biological signals across heterogeneous platforms.

In the revised manuscript, we have supplemented a detailed description of this strategy in the Materials and Methods section, clearly explaining its rationale of using cross-dataset reproducibility as a natural filtering approach to reduce batch confounding. We also explicitly acknowledged the inherent limitation of this design, namely that genuine biological signals masked by strong batch effects might be overlooked. Furthermore, we have noted that future studies should incorporate formal cross-platform normalization and independent experimental validation to further consolidate our findings.

Thank you again for your valuable and constructive comment.

Choice of control group
The study uses obstructive azoospermia (OA) samples as controls. While OA samples are often used in reproductive research, they may not fully represent normal testicular physiology and could introduce confounding factors related to inflammation or other pathological processes. The authors should provide further justification for this choice and discuss its potential impact on the findings.

RE:Thank you very much for your careful comment regarding the choice of control group.

We fully understand your concern. Due to ethical restrictions, it is almost impossible to obtain normal testicular tissue from healthy fertile males for research use. Obstructive azoospermia (OA) is mainly caused by physical obstruction of the sperm outflow tract, with largely preserved spermatogenic function and testicular histological structure. Therefore, OA samples have been widely adopted as a reasonable control in current transcriptomic studies of non-obstructive azoospermia.

Meanwhile, we have frankly discussed the potential limitations of this control selection in the manuscript. Long-term obstruction may lead to elevated epididymal pressure and mild inflammatory responses, which could introduce immune-related confounding factors. Accordingly, the OA group cannot fully represent the physiological immune-privileged status of normal testis, and this may potentially under- or over-estimate the actual differences in certain immune cell subsets.

Notably, another reviewer raised the same concern about the control group selection. We have already supplemented the above justification and potential limitations within the study limitations section of the Discussion in the revised manuscript.

Thank you again for your valuable and constructive suggestion.

Interpretation of drug–gene interaction results
The identification of potential compounds through drug–gene interaction analysis and molecular docking is interesting but appears exploratory. The biological relevance of these predicted interactions in the context of NOA requires further clarification, and the authors should moderate claims regarding therapeutic implications.
RE: Thank you very much for your valuable comment on the interpretation of drug–gene interaction and molecular docking results.

We agree that the compound screening results are purely exploratory. We have substantially revised the relevant descriptions in the revised manuscript.

First, to clarify biological relevance, we have supplemented documented biological links for the key predicted compounds in the Discussion section. This places our computational predictions within a reasonable biological background, rather than presenting them as isolated database screening outputs.

Second, we have moderated all statements regarding therapeutic implications. We explicitly clarify that these findings are hypothesis-generating computational predictions only, intended solely for candidate prioritization and follow-up experimental validation. We have removed any overstated wording that implies imminent clinical translation, ensuring that the conclusions are appropriately restrained and consistent with the exploratory nature of this analysis.

Thank you again for your constructive suggestion.

Minor Comments
The terminology used for the gene signature (e.g., “Hub NOA.Sig”, “Hub NOA.sig”) should be standardized throughout the manuscript.
The Methods section would benefit from clearer descriptions of the machine learning workflow and statistical criteria used for gene selection.
The statistical thresholds used for differential expression analysis should be clarified, including whether multiple testing correction was applied.
Several figures would benefit from more detailed legends to improve readability and interpretation.
Minor grammatical and stylistic revisions are recommended to improve overall clarity.
RE:Thank you very much for your careful and valuable minor comments. We have addressed all the raised points thoroughly in the revised manuscript, as detailed below:

(1) We have carefully checked and standardized the nomenclature of the gene signature throughout the manuscript, uniformly adopting *Hub NOA.Sig* in spelling and capitalization to eliminate inconsistent usage.

(2) In the Methods section, we have reorganized and supplemented the machine learning workflow with detailed descriptions of core hyperparameter settings, consensus criteria for gene selection, and specific procedures for model training and evaluation, making the entire analytical pipeline fully reproducible.

(3) Regarding the statistical thresholds for differential expression analysis, we confirmed that the Benjamini–Hochberg multiple testing correction was indeed applied in the limma analysis. The original statement of “P ≤ 0.05” was a writing error. We have corrected it to adjusted P ≤ 0.05 in the revised manuscript to accurately reflect the analytical process.

(4) We have enriched all figure legends with more detailed explanations to enhance readability and independent interpretability of each graph.

(5) We have conducted a systematic language polishing and stylistic revision across the entire manuscript to fix grammatical issues and improve overall clarity and academic consistency.

Thank you again for your careful review and constructive suggestions.

Summary
Overall, the manuscript addresses an important topic and presents an interesting computational approach to identifying immune-related biomarkers associated with non-obstructive azoospermia. However, clarification of methodological details, stronger discussion of limitations, and more cautious interpretation of computational predictions are needed to improve the rigor and impact of the study.
