# Supplementary material for: Integrated bioinformatics analysis identifies an immune-related gene signature associated with non-obstructive azoospermia
Source: PLoS One. 2026 Oct 12;21(10):e0360305. doi: 10.1371/journal.pone.0360305 (PMC13657319; doi:10.1371/journal.pone.0360305)
Supplement: Supplementary file 4 [file pone.0360305.s009.docx]

**Journal Requirements:**

1. If the reviewer comments include a recommendation to cite specific previously published works, please review and evaluate these publications to determine whether they are relevant and should be cited. There is no requirement to cite these works unless the editor has indicated otherwise. 
RE：Thank you for your careful review and valuable comments.

We appreciate your reminder regarding the standardization of reference citations. We fully understand the core requirement: we should carefully evaluate specific published literatures recommended by reviewers before deciding whether to cite them. After reviewing the current round of review comments, we found that the reviewers did not propose any specific papers to evaluate or cite, nor did the academic editor specify mandatory references to be added. Therefore, no additional references will be supplemented in this revision in response to this general guideline.

We thank you again for your constructive suggestions.
2. Please review your reference list to ensure that it is complete and correct. If you have cited papers that have been retracted, please include the rationale for doing so in the manuscript text, or remove these references and replace them with relevant current references. Any changes to the reference list should be mentioned in the rebuttal letter that accompanies your revised manuscript. If you need to cite a retracted article, indicate the article’s retracted status in the References list and also include a citation and full reference for the retraction notice.
RE：Thank you for your careful review and valuable comments.

We appreciate your reminder on the completeness and accuracy of the reference list. We fully recognize that all cited references must come from reliable sources with accurate bibliographic information, and inappropriate citations of retracted articles should be avoided. Accordingly, we will conduct a thorough item-by-item check of all references during revision. If any retracted papers are identified, we will either explain the rationale for retaining such citations within the main text based on research necessity, or remove these references and replace them with appropriate alternative literatures. All corresponding adjustments to the reference list will be clearly stated in this response letter.

We thank you again for your constructive suggestions.


[Note: HTML markup is below. Please do not edit.]

Reviewers' comments:

Reviewer's Responses to Questions

**Comments to the Author**

1. If the authors have adequately addressed your comments raised in a previous round of review and you feel that this manuscript is now acceptable for publication, you may indicate that here to bypass the “Comments to the Author” section, enter your conflict of interest statement in the “Confidential to Editor” section, and submit your "Accept" recommendation.

Reviewer #1: All comments have been addressed

Reviewer #2: (No Response)

2. Is the manuscript technically sound, and do the data support the conclusions?

The manuscript must describe a technically sound piece of scientific research with data that supports the conclusions. Experiments must have been conducted rigorously, with appropriate controls, replication, and sample sizes. The conclusions must be drawn appropriately based on the data presented.

Reviewer #1: Yes

Reviewer #2: Yes

3. Has the statistical analysis been performed appropriately and rigorously?

Reviewer #1: Yes

Reviewer #2: Yes

4. Have the authors made all data underlying the findings in their manuscript fully available?

The [PLOS Data policy](http://track.editorialmanager.com/CL0/http://www.plosone.org/static/policies.action%23sharing/1/010f019ed3a098be-06dea2f3-8b3c-4bba-b73b-c6b778e1eedb-000000/Wxs5m4CmvanRGDhbpxGGoxqbhtFgFh9qoBClskn3jYM=258) requires authors to make all data underlying the findings described in their manuscript fully available without restriction, with rare exception (please refer to the Data Availability Statement in the manuscript PDF file). The data should be provided as part of the manuscript or its supporting information, or deposited to a public repository. For example, in addition to summary statistics, the data points behind means, medians and variance measures should be available. If there are restrictions on publicly sharing data—e.g. participant privacy or use of data from a third party—those must be specified.

Reviewer #1: Yes

Reviewer #2: Yes

5. Is the manuscript presented in an intelligible fashion and written in standard English?

PLOS ONE does not copyedit accepted manuscripts, so the language in submitted articles must be clear, correct, and unambiguous. Any typographical or grammatical errors should be corrected at revision, so please note any specific errors here.

Reviewer #1: Yes

Reviewer #2: Yes

6. Review Comments to the Author

Please use the space provided to explain your answers to the questions above. You may also include additional comments for the author, including concerns about dual publication, research ethics, or publication ethics. (Please upload your review as an attachment if it exceeds 20,000 characters)

**Reviewer #1:** (No Response)

**Reviewer #2:** Final Opinion from Reviewer #2's Perspective

The revised manuscript demonstrates substantial improvement and successfully addresses the majority of the concerns raised during peer review. The authors have significantly enhanced methodological transparency, strengthened discussions of limitations, and moderated many of their interpretational claims.

The remaining shortcomings primarily involve methodological limitations that cannot be fully corrected retrospectively and a few reporting issues that could be resolved through additional editorial revision. Importantly, none of these residual concerns fundamentally undermine the validity of the analyses or the overall contribution of the work.

Therefore, from Reviewer #2's perspective, the manuscript would most appropriately receive a recommendation of:

Minor Revision

The study is scientifically relevant and methodologically acceptable for publication in PLOS ONE, provided that the authors further clarify the remaining methodological concerns—particularly regarding model performance variability—and continue to ensure that conclusions remain appropriately cautious and proportional to the computational nature of the evidence presented.

The authors are commended for their thorough and rigorous approach to this revision. The major scientific concerns previously raised have been largely and satisfactorily addressed. Specifically, the reframing of causal claims, the transparent discussion of machine-learning limitations, and the acknowledgement of potential batch effects have significantly strengthened the manuscript's academic rigor.

Before the manuscript can be formally accepted for publication in PLOS ONE, the following minor points must be addressed to ensure methodological precision and terminological accuracy:

1. WGCNA Parameter Inconsistency
There remains a contradiction in the text regarding the Weighted Gene Co-expression Network Analysis (WGCNA) parameters. In some sections, the minimum module size is stated as 50, whereas in others, it is listed as 10. Please verify the exact parameter used in your final pipeline and harmonize the text throughout the manuscript to ensure reproducibility.

RE：Thank you for your careful review and valuable comments.

We acknowledge that there exists an inconsistency in the description of the minimum module size for WGCNA throughout the manuscript, which may impair the clarity and reproducibility of our methodological description. We will unify all statements regarding the minimum module size to 50 based on the actual parameters adopted in our final analytical pipeline, and revise the incorrect value of 10 appearing in the Results section to guarantee consistent methodological parameters across the full manuscript.

We thank you again for your constructive suggestions.

2. Control Group Terminology
Please replace all references to "normal testis" or "normal controls" with "OA controls" (Obstructive Azoospermia controls) throughout the text, figures, and tables. Because tissues obtained from OA patients may have altered underlying physiological profiles compared to truly healthy individuals, "OA controls" is the scientifically accurate and standard term for this clinical context.

RE：Thank you for your careful review and valuable comments.

We agree that labeling the control group as "normal testis" or "Normal" throughout the current manuscript lacks precision. Since the control samples in this study were obtained from patients with obstructive azoospermia rather than truly healthy individuals, we will uniformly revise all relevant descriptions of the control group to OA controls (Obstructive Azoospermia controls) across the main text, figures and tables in the revised manuscript, to ensure accurate and consistent terminology.

The detailed revisions are listed below, and all modified contents have been marked in red within the manuscript.

(1) In the Methods section, the phrase "between NOA testis samples and normal testis samples" has been revised to "between NOA testis samples and OA control samples";

(2) In the Results section, the wording "NOA patients and normal testis data" has been changed to "NOA patients and OA control data";

(3) For immune infiltration results, the expression "between the NOA and normal populations" has been modified to "between the NOA and OA control groups";

(4) The column label "Normal" in Figure 2H has been replaced with "OA controls";

(5) The legend label "Normal" in Figure 5A has been revised to "OA controls";

(6) We have conducted a full-text search for all terms used to describe the control group in this manuscript, including "normal testis", "normal controls" and "Normal", and uniformly replaced all of them with "OA controls".

We thank you again for your constructive suggestions.

3. Translational Language in the Abstract
While the main body of the text has been appropriately toned down, the Abstract still contains somewhat over-ambitious translational language. Please further soften these statements in the Abstract to accurately match the balanced, exploratory tone now present in the Discussion section.

RE：Thank you for your careful review and valuable comments.

We acknowledge that several translational statements in the Abstract remain overly assertive, particularly the wording regarding diagnostic optimization, therapeutic repurposing, and clinical management value. Such claims exceed the scope of evidence generated solely from transcriptomic data and computational predictions in this study. We will further soften the relevant descriptions in the revised Abstract, rewriting them to adopt an exploratory, candidate-focused tone that highlights the requirement for subsequent independent validation. This adjustment will align the Abstract’s language with the more cautious, balanced framing adopted within the Discussion section.

The detailed revisions are listed below, and all modified contents have been marked in red within the manuscript.

(1) The phrase “This study aims to identify immune-related biomarkers for improved diagnosis” in the Abstract has been softened. We removed the overstated wording of improved diagnostic value and revised the sentence to focus on exploring molecular features of NOA immunity, reading “explore immune-related molecular signatures associated with NOA”.

(2) The word “robust” in the Abstract’s phrase “to identify a robust immune-derived signature for NOA” has been toned down. This sentence was rewritten as “identify an immune-related gene signature associated with NOA”, to avoid overstating the reliability of computationally screened results.

(3) In the Abstract, the expression “yielded good diagnostic performance with an AUC of 0.87” was adjusted to “yielded good classification performance with an AUC of 0.87”. The AUC value is retained, while direct clinical diagnostic implications are de-emphasized.

(4) The overly assertive statement “potential druggable pairs, suggesting opportunities for therapeutic repurposing” in the Abstract was replaced with a more tentative expression: “candidate drug-gene pairs for subsequent experimental validation”.

(5) The concluding sentence of the Abstract, “This study lays the foundation for mechanism-driven management of male infertility.”, was revised into a more conservative summary as follows: “This work provides transcriptomic clues and candidate molecular targets for future mechanistic investigations into NOA-related male infertility.”

We thank you again for your constructive suggestions.

4. Machine Learning Evaluation Rigor
To provide full transparency regarding the stability of your predictive models, please provide the Confidence Intervals (CIs) or Standard Deviations (SDs) for the Area Under the Curve (AUC) metrics derived from your repeated cross-validation runs. If adding this to the main text is unfeasible, these metrics should be included in a supplementary table.
RE：Thank you for your careful review and valuable comments.

We agree that merely reporting the mean AUC derived from repeated cross-validation fails to fully demonstrate the stability of model performance. In the revised manuscript, we will supplement the mean AUC values, standard deviations (SD), and 95% confidence intervals (CIs) of each machine learning model across 50 rounds of repeated cross-validation, and organize these metrics into a supplementary table to enhance the transparency and reproducibility of our model evaluation results. This table will record indicators including N_resamples, Mean_AUC, SD_AUC, CI95_lower, and CI95_upper for the nb, svmRadialWeights, rf, kknn, and LogitBoost models. Meanwhile, we will briefly interpret the meaning of these stability metrics in the Results section of the main text, so as to avoid evaluating model performance solely based on a single AUC value.

The detailed revisions are listed below, and all modified contents have been marked in red within the manuscript.

We thank you again for your constructive suggestions.
