# Supplementary material for: Integrated bioinformatics analysis identifies an immune-related gene signature associated with non-obstructive azoospermia
Source: PLoS One. 2026 Oct 12;21(10):e0360305. doi: 10.1371/journal.pone.0360305 (PMC13657319; doi:10.1371/journal.pone.0360305)
Supplement: Supplementary file 5 [file pone.0360305.s011.docx]

1. If the authors have adequately addressed your comments raised in a previous round of review and you feel that this manuscript is now acceptable for publication, you may indicate that here to bypass the “Comments to the Author” section, enter your conflict of interest statement in the “Confidential to Editor” section, and submit your "Accept" recommendation.

Reviewer #1: All comments have been addressed

Reviewer #2: All comments have been addressed

**2. Is the manuscript technically sound, and do the data support the conclusions?**

The manuscript must describe a technically sound piece of scientific research with data that supports the conclusions. Experiments must have been conducted rigorously, with appropriate controls, replication, and sample sizes. The conclusions must be drawn appropriately based on the data presented.

Reviewer #1: Yes

Reviewer #2: Yes

**3. Has the statistical analysis been performed appropriately and rigorously?**

Reviewer #1: Yes

Reviewer #2: Yes

**4. Have the authors made all data underlying the findings in their manuscript fully available?**

The [PLOS Data policy](http://track.editorialmanager.com/CL0/http://www.plosone.org/static/policies.action%23sharing/1/010f019fcfd8135f-474e2077-21d7-4d7e-ba94-d6261032d89d-000000/GSWWmXlsxi47HhCKeEoz7YWPs3CIm-vRtH0dHPsDjU8=258) requires authors to make all data underlying the findings described in their manuscript fully available without restriction, with rare exception (please refer to the Data Availability Statement in the manuscript PDF file). The data should be provided as part of the manuscript or its supporting information, or deposited to a public repository. For example, in addition to summary statistics, the data points behind means, medians and variance measures should be available. If there are restrictions on publicly sharing data—e.g. participant privacy or use of data from a third party—those must be specified.

Reviewer #1: Yes

Reviewer #2: Yes

**5. Is the manuscript presented in an intelligible fashion and written in standard English?**

PLOS ONE does not copyedit accepted manuscripts, so the language in submitted articles must be clear, correct, and unambiguous. Any typographical or grammatical errors should be corrected at revision, so please note any specific errors here.

Reviewer #1: (No Response)

Reviewer #2: Yes

**6. Review Comments to the Author**

Please use the space provided to explain your answers to the questions above. You may also include additional comments for the author, including concerns about dual publication, research ethics, or publication ethics. (Please upload your review as an attachment if it exceeds 20,000 characters)

Reviewer #1: (No Response)

Response:

We appreciate the positive evaluation from Reviewer #1. We have carefully revised the manuscript according to the previous review comments. All corresponding modifications have been implemented in the revised manuscript, and the track‑changed version marks all revisions for easy inspection.

Reviewer #2: Reviewer #2 (Second Revision)

I appreciate the authors' careful and thorough revision of the manuscript. The responses provided in the rebuttal letter are clear and comprehensive, and the corresponding changes have been appropriately incorporated into the revised manuscript. My previous concerns have been satisfactorily addressed, as summarized below.

Response:

Thank you very much for your careful review and constructive comments. We highly appreciate your recognition of our revisions. We have addressed all your concerns point‑by‑point as detailed below.

**1. WGCNA Minimum Module Size Inconsistency**

Previous comment: The manuscript contained an inconsistency regarding the minimum module size used for WGCNA, reporting both 50 and 10 in different sections.

Assessment: The revised manuscript now consistently reports a minimum module size of 50 throughout both the Methods (Section 2.3) and Results (Section 3.2). The inconsistency has been fully resolved.

Response:

Thank you for pointing out this inconsistency. We have unified the parameter of minimum module size as 50 in both Section 2.3 (Methods) and Section 3.2 (Results) of the manuscript. The conflicting value “10” has been completely removed to avoid confusion.________________________________________

**2. Control Group Terminology**

Previous comment: References to "normal testis" or "normal controls" should be replaced with "OA controls" throughout the manuscript, figures, and tables to accurately reflect the comparator group.

Assessment: The manuscript now consistently refers to the comparator group as OA controls throughout the Methods, Results, and Discussion. The remaining reference to "normal human testicular tissues" in the Limitations section is appropriate because it specifically refers to truly fertile donor tissue that was unavailable for this study and is clearly distinguished from the OA controls used in the analysis. The authors also indicated that Figure 2H and Figure 5A were updated accordingly; these figure labels should simply be verified in the final production files. Overall, this concern has been satisfactorily addressed.

**Response**: We thank the reviewer for this important suggestion. We have replaced most instances of “normal testis” / “normal controls” with “OA controls” across the main text, tables and figure annotations. As acknowledged, the phrase “normal human testicular tissues” is retained only in the Limitations section, where it describes ideal fertile donor samples that were inaccessible in our present study and is explicitly differentiated from our OA control cohort. Figure 2H and Figure 5A have been revised to adopt consistent terminology, and we will double‑check these labels during the final production proofreading.

Response:

We thank the reviewer for this important suggestion. We have replaced most instances of “normal testis” / “normal controls” with “OA controls” across the main text, tables and figure annotations. As acknowledged, the phrase “normal human testicular tissues” is retained only in the Limitations section, where it describes ideal fertile donor samples that were inaccessible in our present study and is explicitly differentiated from our OA control cohort. Figure 2H and Figure 5A have been revised to adopt consistent terminology, and we will double‑check these labels during the final production proofreading

_______________________________________

**3. Translational Language in the Abstract**

Previous comment: The Abstract contained language that overstated the translational implications of the study and should better reflect the exploratory nature of the findings.

Assessment: The authors have appropriately revised the Abstract. The study objective is now presented in a more exploratory manner, the description of the gene signature has been moderated, "diagnostic performance" has been replaced with "classification performance," and the discussion of drug repurposing has been revised to emphasize candidate drug-gene pairs requiring future experimental validation. The concluding statement has likewise been appropriately tempered. These revisions substantially improve the balance and scientific accuracy of the Abstract.

Response:

We appreciate this valuable comment. We have rewritten the Abstract to highlight the exploratory nature of our bioinformatics analysis. We have moderated statements about the immune‑related gene signature, changed “diagnostic performance” to “classification performance”, adjusted the description of drug repurposing to stress that identified drug‑gene pairs require further experimental validation, and softened the concluding sentence. These edits make the abstract more scientifically balanced and objective.

________________________________________

**4. Machine Learning Evaluation**

Previous comment: Confidence intervals or standard deviations for the reported AUC values should be provided to better characterize model performance during repeated cross-validation.

Assessment: The revised manuscript now reports results from 50 rounds of repeated five-fold cross-validation, including mean AUC, standard deviation, and 95% confidence intervals for all five machine learning classifiers in Supplementary Table S6. The accompanying explanation appropriately notes that these statistics reflect variability from repeated resampling rather than external validation, which is scientifically appropriate. This concern has been fully addressed.

Response:

Thank you for the suggestion. We have supplemented results from 50‑round repeated five‑fold cross‑validation in Supplementary Table S6, including mean AUC values, standard deviations, and 95% confidence intervals for all five machine‑learning classifiers. Corresponding text has been added to clarify that these metrics represent variation from repeated resampling instead of external independent‑set validation.

________________________________________

**Minor Editorial Observation**

I noted one minor wording inconsistency in Section 3.3, where the manuscript states that "six machine learning algorithms" were evaluated, although only five algorithms are listed (Bagged Trees, Bayesian, Boruta, LASSO, and LVQ), consistent with the Methods, Abstract, and Figure 3 legend. This appears to be a typographical error and can be corrected during copyediting.

Response:

Thank you for catching this typographical error. We have revised Section 3.3 and changed “six machine learning algorithms” to “five machine learning algorithms”. The statement is now consistent with the Methods section, Abstract, and Figure 3 legend in the revised manuscript.

________________________________________

**Overall Recommendation**

The authors have addressed all of my previous comments thoroughly and appropriately. The revisions have improved the methodological clarity, terminology, and presentation of the manuscript, and I have no further scientific concerns. I recommend acceptance after correction of the minor editorial issue noted above.

Response:

We sincerely appreciate your thorough review and positive recommendation. We have completed all required revisions including the typo correction mentioned above. All modifications are marked in the version with track changes. We hope the revised manuscript now meets the publication requirements of PLOS ONE.
