# Supplementary material for: Integrated bioinformatics analysis identifies an immune-related gene signature associated with non-obstructive azoospermia
Source: PLoS One. 2026 Oct 12;21(10):e0360305. doi: 10.1371/journal.pone.0360305 (PMC13657319; doi:10.1371/journal.pone.0360305)
Supplement: Peer review history

# Peer review: Decision Letter 0

**DOI:** 10.1371/journal.pone.0360305.r001

**Authors:** Rajakumar Anbazhagan

12 Apr 2026

Dear Dr. Zhong,

Thank you for submitting your manuscript to PLOS ONE. After careful consideration, we feel that it has merit but does not fully meet PLOS ONE’s publication criteria as it currently stands. Therefore, we invite you to submit a revised version of the manuscript that addresses the points raised during the review process.

Please submit your revised manuscript by May 27 2026 11:59PM. If you will need more time than this to complete your revisions, please reply to this message or contact the journal office at plosone@plos.org. When you're ready to submit your revision, log on to https://www.editorialmanager.com/pone/ and select the 'Submissions Needing Revision' folder to locate your manuscript file.

- A letter that responds to each point raised by the academic editor and reviewer(s). You should upload this letter as a separate file labeled 'Response to Reviewers'.
- A marked-up copy of your manuscript that highlights changes made to the original version. You should upload this as a separate file labeled 'Revised Manuscript with Track Changes'.
- An unmarked version of your revised paper without tracked changes. You should upload this as a separate file labeled 'Manuscript'.

If you would like to make changes to your financial disclosure, please include your updated statement in your cover letter. Guidelines for resubmitting your figure files are available below the reviewer comments at the end of this letter.

If applicable, we recommend that you deposit your laboratory protocols in protocols.io to enhance the reproducibility of your results. Protocols.io assigns your protocol its own identifier (DOI) so that it can be cited independently in the future. For instructions see: https://journals.plos.org/plosone/s/submission-guidelines#loc-laboratory-protocols. Additionally, PLOS ONE offers an option for publishing peer-reviewed Lab Protocol articles, which describe protocols hosted on protocols.io. Read more information on sharing protocols at https://plos.org/protocols?utm_medium=editorial-email&utm_source=authorletters&utm_campaign=protocols.

As the corresponding author, your ORCID iD is verified in the submission system and will appear in the published article. PLOS supports the use of ORCID, and we encourage all coauthors to register for an ORCID iD and use it as well. Please encourage your coauthors to verify their ORCID iD within the submission system before final acceptance, as unverified ORCID iDs will not appear in the published article. *Only*  the individual author can complete the verification step; PLOS staff *cannot*  verify ORCID iDs on behalf of authors.

We look forward to receiving your revised manuscript.

Kind regards,

Rajakumar Anbazhagan, Ph. D.

Academic Editor

PLOS One

Journal Requirements:

When submitting your revision, we need you to address these additional requirements.

1. Please ensure that your manuscript meets PLOS ONE's style requirements, including those for file naming. The PLOS ONE style templates can be found at

https://journals.plos.org/plosone/s/file?id=wjVg/PLOSOne_formatting_sample_main_body.pdf and

https://journals.plos.org/plosone/s/file?id=ba62/PLOSOne_formatting_sample_title_authors_affiliations.pdf

2. Please note that PLOS One has specific guidelines on code sharing for submissions in which author-generated code underpins the findings in the manuscript. In these cases, we expect all author-generated code to be made available without restrictions upon publication of the work. Please review our guidelines at https://journals.plos.org/plosone/s/materials-and-software-sharing#loc-sharing-code and ensure that your code is shared in a way that follows best practice and facilitates reproducibility and reuse.

3. PLOS requires an ORCID iD for the corresponding author in Editorial Manager on papers submitted after December 6th, 2016. Please ensure that you have an ORCID iD and that it is validated in Editorial Manager. To do this, go to ‘Update my Information’ (in the upper left-hand corner of the main menu), and click on the Fetch/Validate link next to the ORCID field. This will take you to the ORCID site and allow you to create a new iD or authenticate a pre-existing iD in Editorial Manager.

4. Your ethics statement should only appear in the Methods section of your manuscript. If your ethics statement is written in any section besides the Methods, please move it to the Methods section and delete it from any other section. Please ensure that your ethics statement is included in your manuscript, as the ethics statement entered into the online submission form will not be published alongside your manuscript.

5.  Please include captions for your Supporting Information files at the end of your manuscript, and update any in-text citations to match accordingly. Please see our Supporting Information guidelines for more information: http://journals.plos.org/plosone/s/supporting-information.

6. If the reviewer comments include a recommendation to cite specific previously published works, please review and evaluate these publications to determine whether they are relevant and should be cited. There is no requirement to cite these works unless the editor has indicated otherwise.

[Note: HTML markup is below. Please do not edit.]

Reviewers' comments:

Reviewer's Responses to Questions

**Comments to the Author**

1. Is the manuscript technically sound, and do the data support the conclusions?

Reviewer #1: Partly

Reviewer #2: Partly

**********

2. Has the statistical analysis been performed appropriately and rigorously?

Reviewer #1: Yes

Reviewer #2: No

**********

3. Have the authors made all data underlying the findings in their manuscript fully available?

The PLOS Data policy

Reviewer #1: Yes

Reviewer #2: No

**********

4. Is the manuscript presented in an intelligible fashion and written in standard English?

Reviewer #1: Yes

Reviewer #2: No

**********

Reviewer #1: Reviewer Comments

Summary

This study investigates immune-related biomarkers in non-obstructive azoospermia (NOA) through the analysis of four independent transcriptomic datasets. The authors identify a 12-gene immune-associated signature (Hub NOA. Sig) using an integrative pipeline that combines differential expression analysis, weighted gene co-expression network analysis (WGCNA), and multiple machine learning approaches. The diagnostic performance of this signature is validated across independent cohorts. The study further explores altered immune cell populations, proposes two molecular subtypes, and identifies potential druggable targets through drug–gene interaction analysis and molecular docking.

The manuscript is generally well structured, and the use of multiple datasets along with an integrative analytical approach is a clear strength. The topic is relevant, and the work provides a useful resource for understanding NOA at the transcriptomic level. However, several aspects of the biological interpretation and presentation would benefit from clarification and more cautious framing.

Comments

1. A key limitation of the study is the use of bulk RNA-seq data from heterogeneous testicular tissue. As NOA is associated with significant changes in cellular composition (e.g., germ cell loss), the observed immune signatures may reflect shifts in cell populations or secondary effects rather than primary immune activation.

Suggestion: Although this is mentioned briefly in the discussion, it would strengthen the manuscript to emphasize this limitation more clearly and elaborate on how it may influence the interpretation of immune infiltration results.

2. The use of obstructive azoospermia (OA) samples as controls is understandable given the limited availability of datasets on normal testicular tissue. However, OA does not fully represent normal physiology and may introduce confounding, particularly in immune-related analyses.

Suggestion: Please expand the discussion on how OA differs from normal testicular conditions and how this might affect the interpretation of the results.

3. The manuscript places strong emphasis on immune dysregulation as a driver of NOA. While the identified genes and enrichment analyses support immune involvement, the conclusions at times suggest causality.

Suggestion: Given that the study is based on bulk transcriptomic data, it would be helpful to more clearly distinguish between observed associations and proposed mechanisms. The authors should avoid language suggesting direct immune-mediated damage unless experimentally supported. Framing these findings as hypothesis-generating would improve clarity.

4. The drug-gene interaction and molecular docking analysis is exploratory.

Suggestion: The authors should clearly state that these findings are based on computational predictions. Statements suggesting therapeutic applicability should be toned down, and, if possible, a brief biological rationale linking the identified compounds to NOA could be included.

5. The identification of two molecular subtypes is interesting. However, the manuscript extends this finding to potential applications in personalized treatment.

Suggestion: In the absence of correlations with clinical outcomes, histopathology, or hormonal data, it would be more appropriate to present these subtypes as preliminary transcriptomic classifications. The discussion of clinical applicability should be moderated accordingly.

6. The discussion is quite dense, particularly with gene-by-gene descriptions, and at times leans heavily toward speculative interpretation.

Suggestion: Grouping genes into functional categories may improve readability. It would also be useful to include a brief discussion on whether these immune signatures have been previously reported at the transcript or protein level in NOA studies.

Minor comments:

1. Repetition regarding model robustness and multi-cohort validation could be reduced for better readability.

2. The study workflow should be briefly described in the Results section (as is done in the figure legends). Abbreviations currently listed in the text may be moved to the legend of Figure 1.

3. In Figure 2, the text states that 1,793 genes exhibited differential expression in at least two datasets, whereas Figure 2E shows 1,749. Please ensure consistency.

Recommendation:

Minor revision

The study is methodologically sound and suitable for publication after revisions that improve clarity, moderate interpretation, avoid overstating translational implications, and better align conclusions with the data.

Reviewer #2: Review Comments to the Author

The manuscript presents an integrative bioinformatics analysis to identify immune-related biomarkers associated with non-obstructive azoospermia (NOA). The authors analyze multiple publicly available datasets and apply differential expression analysis, weighted gene co-expression network analysis (WGCNA), and several machine learning approaches to identify a 12-gene immune-related signature associated with NOA. The topic is relevant, and the use of multiple datasets and computational strategies is commendable. However, several methodological and interpretational issues should be addressed to improve the rigor and clarity of the study.

Major Comments

Lack of experimental validation

The study relies entirely on computational analyses of public datasets. While such analyses are valuable for hypothesis generation, the absence of experimental validation limits the strength of the conclusions. Validation of key genes using independent experimental methods (e.g., qPCR, protein expression analysis, or validation in an independent clinical cohort) would significantly strengthen the manuscript.

Machine learning methodology requires clarification

The manuscript employs multiple machine learning algorithms (LASSO, Boruta, LVQ, Bayesian, and Treebag) to identify hub genes; however, the methodological details are insufficient for reproducibility. Important aspects such as hyperparameter settings, feature selection criteria, and details of model optimization are not clearly described. In addition, it is unclear whether nested cross-validation was used to separate feature selection from model evaluation. The authors should provide more detailed methodological descriptions and report variability in model performance (e.g., confidence intervals or standard deviations of AUC values).

Small sample size for model training

The primary training dataset contains a limited number of samples, which may increase the risk of overfitting when applying multiple machine learning algorithms. The authors should discuss this limitation more explicitly and provide additional justification for the robustness of the predictive model.

Dataset heterogeneity and batch effects

The study integrates multiple GEO datasets derived from different platforms. The manuscript does not clearly describe whether batch correction or cross-platform normalization methods were applied. Since batch effects can significantly influence differential expression and downstream analyses, clarification of the preprocessing and normalization steps is necessary.

Choice of control group

The study uses obstructive azoospermia (OA) samples as controls. While OA samples are often used in reproductive research, they may not fully represent normal testicular physiology and could introduce confounding factors related to inflammation or other pathological processes. The authors should provide further justification for this choice and discuss its potential impact on the findings.

Interpretation of drug–gene interaction results

The identification of potential compounds through drug–gene interaction analysis and molecular docking is interesting but appears exploratory. The biological relevance of these predicted interactions in the context of NOA requires further clarification, and the authors should moderate claims regarding therapeutic implications.

Minor Comments

The terminology used for the gene signature (e.g., “Hub NOA.Sig”, “Hub NOA.sig”) should be standardized throughout the manuscript.

The Methods section would benefit from clearer descriptions of the machine learning workflow and statistical criteria used for gene selection.

The statistical thresholds used for differential expression analysis should be clarified, including whether multiple testing correction was applied.

Several figures would benefit from more detailed legends to improve readability and interpretation.

Minor grammatical and stylistic revisions are recommended to improve overall clarity.

Summary

Overall, the manuscript addresses an important topic and presents an interesting computational approach to identifying immune-related biomarkers associated with non-obstructive azoospermia. However, clarification of methodological details, stronger discussion of limitations, and more cautious interpretation of computational predictions are needed to improve the rigor and impact of the study.

**********

what does this mean?). If published, this will include your full peer review and any attached files.

If you choose “no”, your identity will remain anonymous but your review may still be made public.

**Do you want your identity to be public for this peer review?**  For information about this choice, including consent withdrawal, please see our Privacy Policy

Reviewer #1: No

Reviewer #2: **Yes:**  Deepti Bajpai

**********

[NOTE: If reviewer comments were submitted as an attachment file, they will be attached to this email and accessible via the submission site. Please log into your account, locate the manuscript record, and check for the action link "View Attachments". If this link does not appear, there are no attachment files.]

To ensure your figures meet our technical requirements, please review our figure guidelines: https://journals.plos.org/plosone/s/figures

You may also use PLOS’s free figure tool, NAAS, to help you prepare publication quality figures: https://journals.plos.org/plosone/s/figures#loc-tools-for-figure-preparation.

NAAS will assess whether your figures meet our technical requirements by comparing each figure against our figure specifications.

Attachment Submitted filename: PONE-D-26-05936_Reviewer Comments.pdf

# Author comment: Author response to Decision Letter 1

**DOI:** 10.1371/journal.pone.0360305.r002

25 May 2026

Dear Editor,

Your comments, along with those of the other reviewers, are extremely important to our article. We greatly appreciate your insights, which have significantly contributed to enhancing the quality of our work. In this revision, we have prepared a detailed point-by-point response letter for you and the other valued reviewers. This letter acknowledges your feedback and clearly outlines the revisions we have made.

In response to the reviewers' comments, we have made extensive revisions to the manuscript. If there are any further modifications you would like us to consider, we would be more than willing to accommodate them, and we genuinely appreciate your assistance. PLOS ONE is a highly esteemed journal, and we hope that our manuscript can be considered for publication. Thank you very much for your support.

We would like to express our sincere gratitude to you and the reviewers for your valuable comments on our manuscript. In light of the thorough and insightful feedback provided, we have revised the manuscript accordingly. We have added relevant content to address the reviewers' suggestions, and we have rewritten several sections to ensure clarity and completeness. We hope that these modifications meet the reviewers' expectations and enhance the overall quality of the manuscript.

Thank you again sincerely for your time, effort, and kind support.

Best regards,

All authors

Reviewers' comments:

Reviewer's Responses to Questions

Comments to the Author

1. Is the manuscript technically sound, and do the data support the conclusions?

The manuscript must describe a technically sound piece of scientific research with data that supports the conclusions. Experiments must have been conducted rigorously, with appropriate controls, replication, and sample sizes. The conclusions must be drawn appropriately based on the data presented.

Reviewer #1: Partly

Reviewer #2: Partly

2. Has the statistical analysis been performed appropriately and rigorously?

Reviewer #1: Yes

Reviewer #2: No

3. Have the authors made all data underlying the findings in their manuscript fully available?

The PLOS Data policy requires authors to make all data underlying the findings described in their manuscript fully available without restriction, with rare exception (please refer to the Data Availability Statement in the manuscript PDF file). The data should be provided as part of the manuscript or its supporting information, or deposited to a public repository. For example, in addition to summary statistics, the data points behind means, medians and variance measures should be available. If there are restrictions on publicly sharing data—e.g. participant privacy or use of data from a third party—those must be specified.

Reviewer #1: Yes

Reviewer #2: No

4. Is the manuscript presented in an intelligible fashion and written in standard English?

PLOS ONE does not copyedit accepted manuscripts, so the language in submitted articles must be clear, correct, and unambiguous. Any typographical or grammatical errors should be corrected at revision, so please note any specific errors here.

Reviewer #1: Yes

Reviewer #2: No

5. Review Comments to the Author

Please use the space provided to explain your answers to the questions above. You may also include additional comments for the author, including concerns about dual publication, research ethics, or publication ethics. (Please upload your review as an attachment if it exceeds 20,000 characters)

Reviewer #1: Reviewer Comments

Summary

This study investigates immune-related biomarkers in non-obstructive azoospermia (NOA) through the analysis of four independent transcriptomic datasets. The authors identify a 12-gene immune-associated signature (Hub NOA. Sig) using an integrative pipeline that combines differential expression analysis, weighted gene co-expression network analysis (WGCNA), and multiple machine learning approaches. The diagnostic performance of this signature is validated across independent cohorts. The study further explores altered immune cell populations, proposes two molecular subtypes, and identifies potential druggable targets through drug–gene interaction analysis and molecular docking.

The manuscript is generally well structured, and the use of multiple datasets along with an integrative analytical approach is a clear strength. The topic is relevant, and the work provides a useful resource for understanding NOA at the transcriptomic level. However, several aspects of the biological interpretation and presentation would benefit from clarification and more cautious framing.

Comments

1. A key limitation of the study is the use of bulk RNA-seq data from heterogeneous testicular tissue. As NOA is associated with significant changes in cellular composition (e.g., germ cell loss), the observed immune signatures may reflect shifts in cell populations or secondary effects rather than primary immune activation.

Suggestion: Although this is mentioned briefly in the discussion, it would strengthen the manuscript to emphasize this limitation more clearly and elaborate on how it may influence the interpretation of immune infiltration results.

RE: Thank you very much for pointing out this key limitation. We fully agree that the bulk RNA-seq data derived from heterogeneous testicular tissues used in our study introduces cellular composition bias, which is mainly attributed to the massive loss of spermatogenic cells in NOA patients. Accordingly, the identified immune signatures may partially reflect relative proportional changes of cell populations or secondary biological effects, instead of bona fide primary immune activation. This issue directly undermines the reliability of immune infiltration abundance estimation and the subsequent biological interpretation.

In the revised manuscript, we will significantly strengthen the discussion of this limitation. We will elaborate in detail how alterations in cellular composition may overestimate or confound the infiltration levels of specific immune cell subsets. Furthermore, we will remind readers to cautiously interpret the remodeling of the testicular immune microenvironment and the correlations between genes and immune cells, and to fully recognize such confounding effects driven by shifts in cellular proportions. This revision will greatly improve the academic rigor of our study and the robustness of our conclusions.

For the specific details of the changes, please refer to the first item under “Limitations” in the Discussion section; I have marked the revised parts in red font.

Thank you again for your valuable comments and constructive suggestions.

2. The use of obstructive azoospermia (OA) samples as controls is understandable given the limited availability of datasets on normal testicular tissue. However, OA does not fully represent normal physiology and may introduce confounding, particularly in immune-related analyses.

Suggestion: Please expand the discussion on how OA differs from normal testicular conditions and how this might affect the interpretation of the results.

RE:Thank you very much for your careful and constructive comments. We fully agree with this valuable suggestion and have substantially expanded the discussion regarding the potential confounding effects of using obstructive azoospermia (OA) samples as controls in the revised manuscript.

Although employing OA samples as controls represents the most feasible alternative under current public dataset limitations, their potential interference with immune-related interpretations should be interpreted with caution. OA is primarily caused by mechanical obstruction of the reproductive tract, with largely preserved intrinsic spermatogenic function. Nevertheless, long-term elevation of intraluminal pressure and epididymal fluid stasis can retrogradely alter the microenvironment of seminiferous tubules and trigger low-grade aseptic inflammation. Accumulated evidence has confirmed increased focal infiltration of macrophages and lymphocytes in OA testicular tissues, accompanied by upregulated pro-inflammatory cytokines such as IL-6 and TNF-α in epididymal fluid, as well as mild rearrangement of tight junction proteins within the blood-testis barrier.

Accordingly, the observed immune differences between the NOA and OA groups may not entirely reflect primary immune activation inherent to NOA pathogenesis but may partially represent secondary immune perturbations under OA background conditions. Such bias may overestimate the infiltration abundance of immune subsets such as effector memory T cells and regulatory T cells, or misclassify low-grade inflammatory characteristics concomitant with OA as specific immune signatures of NOA. In our analyses, the differential expression of hub genes including CD14 and CXCL16 may genuinely reflect immune microenvironment remodeling in NOA, while also being partially diluted or masked by relative immune activation in the OA control group.

In future studies, incorporating strictly screened testicular tissues from fertile males (e.g., organ donors or individuals undergoing gender reassignment surgery) as external validation controls would help decouple disease-specific immune signatures and further strengthen the objectivity of our conclusions.

We have supplemented detailed descriptions in the Limitations section of the revised manuscript to elaborate that, despite mechanical obstruction as the primary cause of OA, persistent intraductal pressure elevation and microenvironmental disturbance may induce secondary inflammatory responses and immune cell infiltration, substantially deviating the testicular immune landscape from physiological normal status. Supported by existing literature, we further interpret how such deviations may introduce false-positive immune activation signals or overestimate the infiltration levels of effector memory T cells and other immune subsets. This supplementation will guide readers to prudently interpret the observed immune subset alterations. We also acknowledge the necessity of recruiting normal fertile testicular controls for external validation in future work, thereby substantially improving the objectivity and rigor of result interpretation without additional experimental workload.

Thank you again for your insightful comments.

3. The manuscript places strong emphasis on immune dysregulation as a driver of NOA. While the identified genes and enrichment analyses support immune involvement, the conclusions at times suggest causality.

Suggestion: Given that the study is based on bulk transcriptomic data, it would be helpful to more clearly distinguish between observed associations and proposed mechanisms. The authors should avoid language suggesting direct immune-mediated damage unless experimentally supported. Framing these findings as hypothesis-generating would improve clarity.

RE: Thank you very much for your careful and insightful comment. We fully recognize that bulk transcriptomic analysis alone cannot establish causal relationships. We acknowledge that certain statements in the original manuscript may have overinterpreted the results and implicitly suggested a causal role of immune dysregulation in driving NOA pathogenesis.

In the revised manuscript, we have systematically reviewed and revised the entire text. All conclusive and causal claims have been moderated into more conservative association-based descriptions. We have explicitly clarified that the Hub NOA.Sig and related immune-related alterations observed in this study should be regarded as hypothesis-generating findings that require further experimental validation. We have also emphasized that these correlative results merely provide candidate molecular targets and potential research directions for subsequent mechanistic exploration, rather than demonstrating direct immune-mediated pathogenic damage. This revision greatly improves the precision, clarity and scientific rigor of our conclusions.

Thank you again for your valuable suggestion.

4. The drug-gene interaction and molecular docking analysis is exploratory.

Suggestion: The authors should clearly state that these findings are based on computational predictions. Statements suggesting therapeutic applicability should be toned down, and, if possible, a brief biological rationale linking the identified compounds to NOA could be included.

RE: Thank you very much for your thoughtful comment. We fully agree that our drug–gene interaction screening and molecular docking analyses are essentially exploratory computational predictions, and we acknowledge that some descriptions implying direct therapeutic applicability should be appropriately moderated.

In the revised manuscript, we have thoroughly revised this section. We have explicitly clarified that all relevant results are purely computational predictions derived from bioinformatics analysis. We have deliberately toned down overstated claims and framed these compounds only as preliminary candidates obtained from in silico screening, while removing any wording that suggests immediate clinical or therapeutic potential. Furthermore, we have supplemented the Discussion with a brief biological rationale linking loperamide, acitretin and retinol to testicular physiology, immune regulation and spermatogenesis homeostasis, to better contextualize these computational predictions. These revisions make the exploratory findings more objective, cautious, and scientifically rigorous.

Thank you again for your valuable suggestion.

5. The identification of two molecular subtypes is interesting. However, the manuscript extends this finding to potential applications in personalized treatment.

Suggestion: In the absence of correlations with clinical outcomes, histopathology, or hormonal data, it would be more appropriate to present these subtypes as preliminary transcriptomic classifications. The discussion of clinical applicability should be moderated accordingly.

RE:Thank you very much for your careful and insightful review. We fully agree that it is premature to directly extend the identified two molecular subtypes to personalized treatment applications without valid correlations with clinical outcomes, histopathological features, and hormonal profiles.

In the revised manuscript, we have rigorously adjusted the relevant descriptions. We now explicitly define these two subtypes as preliminary transcriptomic classifications. We have appropriately moderated any overinterpretation regarding clinical applicability and therapeutic guiding value, and refrain from making direct clinical inferences. Meanwhile, we clarify that these molecular subtypes merely serve as a hypothesis-generating framework to provide exploratory directions for future studies integrating clinical phenotypic data. Such revisions ensure that our conclusions are fully consistent with the available evidence base.

Thank you again for your valuable and constructive comment.

6. The discussion is quite dense, particularly with gene-by-gene descriptions, and at times leans heavily toward speculative interpretation.

Suggestion: Grouping genes into functional categories may improve readability. It would also be useful to include a brief discussion on whether these immune signatures have been previously reported at the transcript or protein level in NOA studies.

RE:Thank you very much for your careful and valuable comments. We fully agree that the current Discussion section is relatively dense with gene-by-gene descriptions and occasionally contains overly speculative interpretations.

In the revised manuscript, we have reorganized these 12 hub genes into several functional categories, such as innate immunity and pattern recognition, chemokine and Th2 signaling, as well as stress and metabolic immune regulation. This grouping greatly improves the logical structure and readability of the discussion. In addition, we have supplemented each functional module with existing evidence from previous NOA studies, summarizing relevant transcript-level and protein-level reports of these immune signatures in the literature. This arrangement balances speculative interpretation with published experimental evidence, making the Discussion more concise, objective, and evidence-based.

Thank you again for your constructive suggestion.

Minor comments:

1. Repetition regarding model robustness and multi-cohort validation could be reduced for better readability.

2. The study workflow should be briefly described in the Results section (as is done in the figure legends). Abbreviations currently listed in the text may be moved to the legend of Figure 1.

3. In Figure 2, the text states that 1,793 genes exhibited differential expression in at least two datasets, whereas Figure 2E shows 1,749. Please ensure consistency.

RE:Thank you very much for your careful comments and valuable suggestions.

1.We have carefully checked the full manuscript and appropriately reduced repetitive descriptions regarding model robustness and multi-cohort validation. We only retain key information at essential positions in the Results and Discussion sections to improve overall readability and avoid redundant narration.

2. We have followed your suggestion and briefly summarized the overall study workflow at the beginning of the Results section. Meanwhile, all abbreviations originally explained in the main text have been moved to the legend of Figure 1 to simplify the body text layout and standardize manuscript writing.

3. We have carefully checked the numerical inconsistency between the main text and Figure 2E. The correct number has been unified and revised to ensure complete consistency between textual description and graphical presentation.

Thank you again for your thoughtful comments, which greatly help improve the clarity and standardization of our manuscript.

Recommendation:

Minor revision

The study is methodologically sound and suitable for publication after revisions that improve clarity, moderate interpretation, avoid overstating translational implications, and better align conclusions with the data.

Reviewer #2: Review Comments to the Author

The manuscript presents an integrative bioinformatics analysis to identify immune-related biomarkers associated with non-obstructive azoospermia (NOA). The authors analyze multiple publicly available datasets and apply differential expression analysis, weighted gene co-expression network analysis (WGCNA), and several machine learning approaches to identify a 12-gene immune-related signature associated with NOA. The topic is relevant, and the use of multiple datasets and computational strategies is commendable. However, several methodological and interpretational issues should be addressed to improve the rigor and clarity of the study.

Major Comments

Lack of experimental validation

The study relies entirely on computational analyses of public datasets. While such analyses are valuable for hypothesis generation, the absence of experimental validation limits the strength of the conclusions. Validation of key genes using independent experimental methods (e.g., qPCR, protein expression analysis, or validation in an independent clinical cohort) would significantly strengthen the manuscript.

RE:Thank you very much for your valuable comment. We fully acknowledge that this study is entirely based on computational analysis of public datasets, and the lack of independent experimental validation inevitably limits the robustness of our conclusions.

In the revised manuscript, we have explicitly emphasized this limitation in the Discussion section. We clearly state that the present work is hypothesis-generating exploratory research, and highlight that the identified Hub NOA.Sig genes, altered immune cell profiles, and molecular subtype classification all require further experimental verification, such as qPCR detection, protein expression analysis, or validation in an independent clinical cohort, before being regarded as definitive conclusions. We also specify concrete directions for subsequent experimental validation for each core finding, to properly interpret the reliability of current results and guide future study design.

We have already supplemented relevant statements in the Discussion section according to your suggestion in the revised version.

Thank you again for your constructive and insightful comment.

Machine learning methodology requires clarification

The manuscript employs multiple machine learning algorithms (LASSO, Boruta, LVQ, Bayesian, and Treebag) to identify hub genes; however, the methodological details are insufficient for reproducibility. Important aspects such as hyperparameter settings, feature selection criteria, and details of model optimization are not clearly described. In addition, it is unclear whether nested cross-validation was used to separate feature selection from model evaluation. The authors should provide more detailed methodological descriptions and report variability in model performance (e.g., confidence intervals or standard deviations of AUC values).

RE:Thank you very much for your careful and constructive comments. We fully agree that the machine learning methodology needs further clarification to improve methodological transparency and reproducibility.

First, we have supplemented detailed methodological descriptions in the Materials and Methods section regarding hyperparameter settings, feature selection criteria, and model optimization procedures for all five algorithms (LASSO, Boruta, LVQ, Bayesian, and Treebag). We have specified the R packages, iteration settings, cross‑validation strategies, key hyperparameters, and gene screening thresholds used in each algorithm, making the entire feature selection workflow fully reproducible.

Second, we acknowledge that nested cross‑validation was not adopted to separate feature selection from model evaluation in the current study. We have frankly stated this methodological limitation in the Discussion section. We clarified that this arrangement may introduce information leakage and optimistic bias, which could overestimate model generalization performance on external validation cohorts. Readers are reminded to interpret the diagnostic efficacy cautiously, and further validation in completely independent clinical cohorts is recommended in future work.

Third, we fully understand the importance of reporting performance variability such as AUC confidence intervals or standard deviations. Nevertheless, limited sample size in the training set, small fold‑wise validation subsets, and non‑nested feature selection make it inappropriate to directly calculate and report AUC variability metrics, which may produce misleading estimates rather than reflecting true generalization error. Therefore, we have explained this methodological consideration explicitly in the Discussion. Instead of forcing unreliable variability statistics, we use consistent performance across multiple independent GEO cohorts to reflect model robustness, and note that future larger‑sample or nested resampling designs are required for reliable estimation of performance variability.

We have completed all the above revisions and additions in the revised manuscript as suggested. Thank you again for your valuable comments, which greatly improve the methodological rigor and interpretability of our work.

Small sample size for model training

The primary training dataset contains a limited number of samples, which may increase the risk of overfitting when applying multiple machine learning algorithms. The authors should discuss this limitation more explicitly and provide additional justification for the robustness of the predictive model.

RE:Thank you very much for pointing out this limitation. We acknowledge that the limited sample size of the primary training dataset may increase the risk of overfitting when using multiple machine learning algorithms. We have added a concise explanation in the Discussion to explicitly state this concern, and meanwhile summarized multi-cohort validation evidence to justify the robustness of the model.

Thank you again for your constructive suggestion.

Dataset heterogeneity and batch effects

The study integrates multiple GEO datasets derived from different platforms. The manuscript does not clearly describe whether batch correction or cross-platform normalization methods were applied. Since batch effects can significantly influence differential expression and downstream analyses, clarification of the preprocessing and normalization steps is necessary.

RE:Thank you very much for raising this important methodological concern regarding dataset heterogeneity and batch effects. We have carefully checked our analytical pipeline and confirm that no dedicated batch correction or cross-platform normalization algorithms were applied in this study.

Instead, we adopted a consensus strategy: differential expression analysis was performed independently for each GEO dataset. We then integrated genes that were consistently differentially expressed in at least two datasets to filter robust biological signals across heterogeneous platforms.

In the revised manuscript, we have supplemented a detailed description of this strategy in the Materials and Methods section, clearly explaining its rationale of using cross-dataset reproducibility as a natural filtering approach to reduce batch confounding. We also explicitly acknowledged the inherent limitation of this design, namely that genuine biological signals masked by strong batch effects might be overlooked. Furthermore, we have noted that future studies should incorporate formal cross-platform normalization and independent experimental validation to further consolidate our findings.

Thank you again for your valuable and constructive comment.

Choice of control group

The study uses obstructive azoospermia (OA) samples as controls. While OA samples are often used in reproductive research, they may not fully represent normal testicular physiology and could introduce confounding factors related to inflammation or other pathological processes. The authors should provide further justification for this choice and discuss its potential impact on the findings.

RE:Thank you very much for your careful comment regarding the choice of control group.

We fully understand your concern. Due to ethical restrictions, it is almost impossible to obtain normal testicular tissue from healthy fertile males for research use. Obstructive azoospermia (OA) is mainly caused by physical obstruction of the sperm outflow tract, with largely preserved spermatogenic function and testicular histological structure. Therefore, OA samples have been widely adopted as a reasonable control in current transcriptomic studies of non-obstructive azoospermia.

Meanwhile, we have frankly discussed the potential limitations of this control selection in the manuscript. Long-term obstruction may lead to elevated epididymal pressure and mild inflammatory responses, which could introduce immune-related confounding factors. Accordingly, the OA group cannot fully represent the physiological immune-privileged status of normal testis, and this may potentially under- or over-estimate the actual differences in certain immune cell subsets.

Notably, another reviewer raised the same concern about the control group selection. We have already supplemented the above justification and potential limitations within the study limitations section of the Discussion in the revised manuscript.

Thank you again for your valuable and constructive suggestion.

Interpretation of drug–gene interaction results

The identification of potential compounds through drug–gene interaction analysis and molecular docking is interesting but appears exploratory. The biological relevance of these predicted interactions in the context of NOA requires further clarification, and the authors should moderate claims regarding therapeutic implications.

RE: Thank you very much for your valuable comment on the interpretation of drug–gene interaction and molecular docking results.

We agree that the compound screening results are purely exploratory. We have substantially revised the relevant descriptions in the revised manuscript.

First, to clarify biological relevance, we have supplemented documented biological links for the key predicted compounds in the Discussion section. This places our computational predictions within a reasonable biological background, rather than presenting them as isolated database screening outputs.

Second, we have moderated all statements regarding therapeutic implications. We explicitly clarify that these findings are hypothesis-generating computational predictions only, intended solely for candidate prioritization and follow-up experimental validation. We have removed any overstated wording that implies imminent clinical translation, ensuring that the conclusions are appropriately restrained and consistent with the exploratory nature of this analysis.

Thank you again for your constructive suggestion.

Minor Comments

The terminology used for the gene signature (e.g., “Hub NOA.Sig”, “Hub NOA.sig”) should be standardized throughout the manuscript.

The Methods section would benefit from clearer descriptions of the machine learning workflow and statistical criteria used for gene selection.

The statistical thresholds used for differential expression analysis should be clarified, including whether multiple testing correction was applied.

Several figures would benefit from more detailed legends to improve readability and interpretation.

Minor grammatical and stylistic revisions are recommended to improve overall clarity.

RE:Thank you very much for your careful and valuable minor comments. We have addressed all the raised points thoroughly in the revised manuscript, as detailed below:

(1) We have carefully checked and standardized the nomenclature of the gene signature throughout the manuscript, uniformly adopting Hub NOA.Sig in spelling and capitalization to eliminate inconsistent usage.

(2) In the Methods section, we have reorganized and supplemented the machine learning workflow with detailed descriptions of core hyperparameter settings, consensus criteria for gene selection, and specific procedures for model training and evaluation, making the entire analytical pipeline fully reproducible.

(3) Regarding the statistical thresholds for differential expression analysis, we confirmed that the Benjamini–Hochberg multiple testing correction was indeed applied in the limma analysis. The original statement of “P ≤ 0.05” was a writing error. We have corrected it to adjusted P ≤ 0.05 in the revised manuscript to accurately reflect the analytical process.

(4) We have enriched all figure legends with more detailed explanations to enhance readability and independent interpretability of each graph.

(5) We have conducted a systematic language polishing and stylistic revision across the entire manuscript to fix grammatical issues and improve overall clarity and academic consistency.

Thank you again for your careful review and constructive suggestions.

Summary

Overall, the manuscript addresses an important topic and presents an interesting computational approach to identifying immune-related biomarkers associated with non-obstructive azoospermia. However, clarification of methodological details, stronger discussion of limitations, and more cautious interpretation of computational predictions are needed to improve the rigor and impact of the study.

Attachment Submitted filename: Response to reviewers.docx

# Peer review: Decision Letter 1

**DOI:** 10.1371/journal.pone.0360305.r003

**Authors:** Rajakumar Anbazhagan

16 Jun 2026

Dear Dr. Zhong,

Thank you for submitting your manuscript to PLOS ONE. After careful consideration, we feel that it has merit but does not fully meet PLOS ONE’s publication criteria as it currently stands. Therefore, we invite you to submit a revised version of the manuscript that addresses the points raised during the review process.

Please submit your revised manuscript by Jul 31 2026 11:59PM. If you will need more time than this to complete your revisions, please reply to this message or contact the journal office at plosone@plos.org. When you're ready to submit your revision, log on to https://www.editorialmanager.com/pone/ and select the 'Submissions Needing Revision' folder to locate your manuscript file.

- A letter that responds to each point raised by the academic editor and reviewer(s). You should upload this letter as a separate file labeled 'Response to Reviewers'.
- A marked-up copy of your manuscript that highlights changes made to the original version. You should upload this as a separate file labeled 'Revised Manuscript with Track Changes'.
- An unmarked version of your revised paper without tracked changes. You should upload this as a separate file labeled 'Manuscript'.

If you would like to make changes to your financial disclosure, please include your updated statement in your cover letter. Guidelines for resubmitting your figure files are available below the reviewer comments at the end of this letter.

If applicable, we recommend that you deposit your laboratory protocols in protocols.io to enhance the reproducibility of your results. Protocols.io assigns your protocol its own identifier (DOI) so that it can be cited independently in the future. For instructions see: https://journals.plos.org/plosone/s/submission-guidelines#loc-laboratory-protocols. Additionally, PLOS ONE offers an option for publishing peer-reviewed Lab Protocol articles, which describe protocols hosted on protocols.io. Read more information on sharing protocols at https://plos.org/protocols?utm_medium=editorial-email&utm_source=authorletters&utm_campaign=protocols.

As the corresponding author, your ORCID iD is verified in the submission system and will appear in the published article. PLOS supports the use of ORCID, and we encourage all coauthors to register for an ORCID iD and use it as well. Please encourage your coauthors to verify their ORCID iD within the submission system before final acceptance, as unverified ORCID iDs will not appear in the published article. *Only*  the individual author can complete the verification step; PLOS staff *cannot*  verify ORCID iDs on behalf of authors.

We look forward to receiving your revised manuscript.

Kind regards,

Rajakumar Anbazhagan, Ph. D.

Academic Editor

PLOS One

**Journal Requirements:**

1. If the reviewer comments include a recommendation to cite specific previously published works, please review and evaluate these publications to determine whether they are relevant and should be cited. There is no requirement to cite these works unless the editor has indicated otherwise.

2. Please review your reference list to ensure that it is complete and correct. If you have cited papers that have been retracted, please include the rationale for doing so in the manuscript text, or remove these references and replace them with relevant current references. Any changes to the reference list should be mentioned in the rebuttal letter that accompanies your revised manuscript. If you need to cite a retracted article, indicate the article’s retracted status in the References list and also include a citation and full reference for the retraction notice.

[Note: HTML markup is below. Please do not edit.]

Reviewers' comments:

Reviewer's Responses to Questions

**Comments to the Author**

Reviewer #1: All comments have been addressed

Reviewer #2: (No Response)

**********

2. Is the manuscript technically sound, and do the data support the conclusions?

Reviewer #1: Yes

Reviewer #2: Yes

**********

3. Has the statistical analysis been performed appropriately and rigorously?

Reviewer #1: Yes

Reviewer #2: Yes

**********

4. Have the authors made all data underlying the findings in their manuscript fully available?

The PLOS Data policy

Reviewer #1: Yes

Reviewer #2: Yes

**********

5. Is the manuscript presented in an intelligible fashion and written in standard English?

Reviewer #1: Yes

Reviewer #2: Yes

**********

**Reviewer #1:**  (No Response)

**Reviewer #2:**  Final Opinion from Reviewer #2's Perspective

The revised manuscript demonstrates substantial improvement and successfully addresses the majority of the concerns raised during peer review. The authors have significantly enhanced methodological transparency, strengthened discussions of limitations, and moderated many of their interpretational claims.

The remaining shortcomings primarily involve methodological limitations that cannot be fully corrected retrospectively and a few reporting issues that could be resolved through additional editorial revision. Importantly, none of these residual concerns fundamentally undermine the validity of the analyses or the overall contribution of the work.

Therefore, from Reviewer #2's perspective, the manuscript would most appropriately receive a recommendation of:

Minor Revision

The study is scientifically relevant and methodologically acceptable for publication in PLOS ONE, provided that the authors further clarify the remaining methodological concerns—particularly regarding model performance variability—and continue to ensure that conclusions remain appropriately cautious and proportional to the computational nature of the evidence presented.

The authors are commended for their thorough and rigorous approach to this revision. The major scientific concerns previously raised have been largely and satisfactorily addressed. Specifically, the reframing of causal claims, the transparent discussion of machine-learning limitations, and the acknowledgement of potential batch effects have significantly strengthened the manuscript's academic rigor.

Before the manuscript can be formally accepted for publication in PLOS ONE, the following minor points must be addressed to ensure methodological precision and terminological accuracy:

1. WGCNA Parameter Inconsistency

There remains a contradiction in the text regarding the Weighted Gene Co-expression Network Analysis (WGCNA) parameters. In some sections, the minimum module size is stated as 50, whereas in others, it is listed as 10. Please verify the exact parameter used in your final pipeline and harmonize the text throughout the manuscript to ensure reproducibility.

2. Control Group Terminology

Please replace all references to "normal testis" or "normal controls" with "OA controls" (Obstructive Azoospermia controls) throughout the text, figures, and tables. Because tissues obtained from OA patients may have altered underlying physiological profiles compared to truly healthy individuals, "OA controls" is the scientifically accurate and standard term for this clinical context.

3. Translational Language in the Abstract

While the main body of the text has been appropriately toned down, the Abstract still contains somewhat over-ambitious translational language. Please further soften these statements in the Abstract to accurately match the balanced, exploratory tone now present in the Discussion section.

4. Machine Learning Evaluation Rigor

To provide full transparency regarding the stability of your predictive models, please provide the Confidence Intervals (CIs) or Standard Deviations (SDs) for the Area Under the Curve (AUC) metrics derived from your repeated cross-validation runs. If adding this to the main text is unfeasible, these metrics should be included in a supplementary table.

**********

what does this mean?). If published, this will include your full peer review and any attached files.

If you choose “no”, your identity will remain anonymous but your review may still be made public.

**Do you want your identity to be public for this peer review?**  For information about this choice, including consent withdrawal, please see our Privacy Policy

Reviewer #1: No

Reviewer #2: No

**********

[NOTE: If reviewer comments were submitted as an attachment file, they will be attached to this email and accessible via the submission site. Please log into your account, locate the manuscript record, and check for the action link "View Attachments". If this link does not appear, there are no attachment files.]

To ensure your figures meet our technical requirements, please review our figure guidelines: https://journals.plos.org/plosone/s/figures

You may also use PLOS’s free figure tool, NAAS, to help you prepare publication quality figures: https://journals.plos.org/plosone/s/figures#loc-tools-for-figure-preparation.

NAAS will assess whether your figures meet our technical requirements by comparing each figure against our figure specifications.

# Author comment: Author response to Decision Letter 2

**DOI:** 10.1371/journal.pone.0360305.r004

17 Jul 2026

Journal Requirements:

1. If the reviewer comments include a recommendation to cite specific previously published works, please review and evaluate these publications to determine whether they are relevant and should be cited. There is no requirement to cite these works unless the editor has indicated otherwise.

RE：Thank you for your careful review and valuable comments.

We appreciate your reminder regarding the standardization of reference citations. We fully understand the core requirement: we should carefully evaluate specific published literatures recommended by reviewers before deciding whether to cite them. After reviewing the current round of review comments, we found that the reviewers did not propose any specific papers to evaluate or cite, nor did the academic editor specify mandatory references to be added. Therefore, no additional references will be supplemented in this revision in response to this general guideline.

We thank you again for your constructive suggestions.

2. Please review your reference list to ensure that it is complete and correct. If you have cited papers that have been retracted, please include the rationale for doing so in the manuscript text, or remove these references and replace them with relevant current references. Any changes to the reference list should be mentioned in the rebuttal letter that accompanies your revised manuscript. If you need to cite a retracted article, indicate the article’s retracted status in the References list and also include a citation and full reference for the retraction notice.

RE：Thank you for your careful review and valuable comments.

We appreciate your reminder on the completeness and accuracy of the reference list. We fully recognize that all cited references must come from reliable sources with accurate bibliographic information, and inappropriate citations of retracted articles should be avoided. Accordingly, we will conduct a thorough item-by-item check of all references during revision. If any retracted papers are identified, we will either explain the rationale for retaining such citations within the main text based on research necessity, or remove these references and replace them with appropriate alternative literatures. All corresponding adjustments to the reference list will be clearly stated in this response letter.

We thank you again for your constructive suggestions.

[Note: HTML markup is below. Please do not edit.]

Reviewers' comments:

Reviewer's Responses to Questions

Comments to the Author

1. If the authors have adequately addressed your comments raised in a previous round of review and you feel that this manuscript is now acceptable for publication, you may indicate that here to bypass the “Comments to the Author” section, enter your conflict of interest statement in the “Confidential to Editor” section, and submit your "Accept" recommendation.

Reviewer #1: All comments have been addressed

Reviewer #2: (No Response)

2. Is the manuscript technically sound, and do the data support the conclusions?

The manuscript must describe a technically sound piece of scientific research with data that supports the conclusions. Experiments must have been conducted rigorously, with appropriate controls, replication, and sample sizes. The conclusions must be drawn appropriately based on the data presented.

Reviewer #1: Yes

Reviewer #2: Yes

3. Has the statistical analysis been performed appropriately and rigorously?

Reviewer #1: Yes

Reviewer #2: Yes

4. Have the authors made all data underlying the findings in their manuscript fully available?

The PLOS Data policy requires authors to make all data underlying the findings described in their manuscript fully available without restriction, with rare exception (please refer to the Data Availability Statement in the manuscript PDF file). The data should be provided as part of the manuscript or its supporting information, or deposited to a public repository. For example, in addition to summary statistics, the data points behind means, medians and variance measures should be available. If there are restrictions on publicly sharing data—e.g. participant privacy or use of data from a third party—those must be specified.

Reviewer #1: Yes

Reviewer #2: Yes

5. Is the manuscript presented in an intelligible fashion and written in standard English?

PLOS ONE does not copyedit accepted manuscripts, so the language in submitted articles must be clear, correct, and unambiguous. Any typographical or grammatical errors should be corrected at revision, so please note any specific errors here.

Reviewer #1: Yes

Reviewer #2: Yes

6. Review Comments to the Author

Please use the space provided to explain your answers to the questions above. You may also include additional comments for the author, including concerns about dual publication, research ethics, or publication ethics. (Please upload your review as an attachment if it exceeds 20,000 characters)

Reviewer #1: (No Response)

Reviewer #2: Final Opinion from Reviewer #2's Perspective

The revised manuscript demonstrates substantial improvement and successfully addresses the majority of the concerns raised during peer review. The authors have significantly enhanced methodological transparency, strengthened discussions of limitations, and moderated many of their interpretational claims.

The remaining shortcomings primarily involve methodological limitations that cannot be fully corrected retrospectively and a few reporting issues that could be resolved through additional editorial revision. Importantly, none of these residual concerns fundamentally undermine the validity of the analyses or the overall contribution of the work.

Therefore, from Reviewer #2's perspective, the manuscript would most appropriately receive a recommendation of:

Minor Revision

The study is scientifically relevant and methodologically acceptable for publication in PLOS ONE, provided that the authors further clarify the remaining methodological concerns—particularly regarding model performance variability—and continue to ensure that conclusions remain appropriately cautious and proportional to the computational nature of the evidence presented.

The authors are commended for their thorough and rigorous approach to this revision. The major scientific concerns previously raised have been largely and satisfactorily addressed. Specifically, the reframing of causal claims, the transparent discussion of machine-learning limitations, and the acknowledgement of potential batch effects have significantly strengthened the manuscript's academic rigor.

Before the manuscript can be formally accepted for publication in PLOS ONE, the following minor points must be addressed to ensure methodological precision and terminological accuracy:

1. WGCNA Parameter Inconsistency

There remains a contradiction in the text regarding the Weighted Gene Co-expression Network Analysis (WGCNA) parameters. In some sections, the minimum module size is stated as 50, whereas in others, it is listed as 10. Please verify the exact parameter used in your final pipeline and harmonize the text throughout the manuscript to ensure reproducibility.

RE：Thank you for your careful review and valuable comments.

We acknowledge that there exists an inconsistency in the description of the minimum module size for WGCNA throughout the manuscript, which may impair the clarity and reproducibility of our methodological description. We will unify all statements regarding the minimum module size to 50 based on the actual parameters adopted in our final analytical pipeline, and revise the incorrect value of 10 appearing in the Results section to guarantee consistent methodological parameters across the full manuscript.

We thank you again for your constructive suggestions.

2. Control Group Terminology

Please replace all references to "normal testis" or "normal controls" with "OA controls" (Obstructive Azoospermia controls) throughout the text, figures, and tables. Because tissues obtained from OA patients may have altered underlying physiological profiles compared to truly healthy individuals, "OA controls" is the scientifically accurate and standard term for this clinical context.

RE：Thank you for your careful review and valuable comments.

We agree that labeling the control group as "normal testis" or "Normal" throughout the current manuscript lacks precision. Since the control samples in this study were obtained from patients with obstructive azoospermia rather than truly healthy individuals, we will uniformly revise all relevant descriptions of the control group to OA controls (Obstructive Azoospermia controls) across the main text, figures and tables in the revised manuscript, to ensure accurate and consistent terminology.

The detailed revisions are listed below, and all modified contents have been marked in red within the manuscript.

(1) In the Methods section, the phrase "between NOA testis samples and normal testis samples" has been revised to "between NOA testis samples and OA control samples";

(2) In the Results section, the wording "NOA patients and normal testis data" has been changed to "NOA patients and OA control data";

(3) For immune infiltration results, the expression "between the NOA and normal populations" has been modified to "between the NOA and OA control groups";

(4) The column label "Normal" in Figure 2H has been replaced with "OA controls";

(5) The legend label "Normal" in Figure 5A has been revised to "OA controls";

(6) We have conducted a full-text search for all terms used to describe the control group in this manuscript, including "normal testis", "normal controls" and "Normal", and uniformly replaced all of them with "OA controls".

We thank you again for your constructive suggestions.

3. Translational Language in the Abstract

While the main body of the text has been appropriately toned down, the Abstract still contains somewhat over-ambitious translational language. Please further soften these statements in the Abstract to accurately match the balanced, exploratory tone now present in the Discussion section.

RE：Thank you for your careful review and valuable comments.

We acknowledge that several translational statements in the Abstract remain overly assertive, particularly the wording regarding diagnostic optimization, therapeutic repurposing, and clinical management value. Such claims exceed the scope of evidence generated solely from transcriptomic data and computational predictions in this study. We will further soften the relevant descriptions in the revised Abstract, rewriting them to adopt an exploratory, candidate-focused tone that highlights the requirement for subsequent independent validation. This adjustment will align the Abstract’s language with the more cautious, balanced framing adopted within the Discussion section.

The detailed revisions are listed below, and all modified contents have been marked in red within the manuscript.

(1) The phrase “This study aims to identify immune-related biomarkers for improved diagnosis” in the Abstract has been softened. We removed the overstated wording of improved diagnostic value and revised the sentence to focus on exploring molecular features of NOA immunity, reading “explore immune-related molecular signatures associated with NOA”.

(2) The word “robust” in the Abstract’s phrase “to identify a robust immune-derived signature for NOA” has been toned down. This sentence was rewritten as “identify an immune-related gene signature associated with NOA”, to avoid overstating the reliability of computationally screened results.

(3) In the Abstract, the expression “yielded good diagnostic performance with an AUC of 0.87” was adjusted to “yielded good classification performance with an AUC of 0.87”. The AUC value is retained, while direct clinical diagnostic implications are de-emphasized.

(4) The overly assertive statement “potential druggable pairs, suggesting opportunities for therapeutic repurposing” in the Abstract was replaced with a more tentative expression: “candidate drug-gene pairs for subsequent experimental validation”.

(5) The concluding sentence of the Abstract, “This study lays the foundation for mechanism-driven management of male infertility.”, was revised into a more conservative summary as follows: “This work provides transcriptomic clues and candidate molecular targets for future mechanistic investigations into NOA-related male infertility.”

We thank you again for your constructive suggestions.

4. Machine Learning Evaluation Rigor

To provide full transparency regarding the stability of your predictive models, please provide the Confidence Intervals (CIs) or Standard Deviations (SDs) for the Area Under the Curve (AUC) metrics derived from your repeated cross-validation runs. If adding this to the main text is unfeasible, these metrics should be included in a supplementary table.

RE：Thank you for your careful review and valuable comments.

We agree that merely reporting the mean AUC derived from repeated cross-validation fails to fully demonstrate the stability of model performance. In the revised manuscript, we will supplement the mean AUC values, standard deviations (SD), and 95% confidence intervals (CIs) of each machine learning model across 50 rounds of repeated cross-validation, and organize these metrics into a supplementary table to enhance the transparency and reproducibility of our model evaluation results. This table will record indicators including N_resamples, Mean_AUC, SD_AUC, CI95_lower, and CI95_upper for the nb, svmRadialWeights, rf, kknn, and LogitBoost models. Meanwhile, we will briefly interpret the meaning of these stability metrics in the Results section of the main text, so as to avoid evaluating model performance solely based on a single AUC value.

The detailed revisions are listed below, and all modified contents have been marked in red within the manuscript.

We thank you again for your constructive suggestions.

Attachment Submitted filename: Response_to_reviewers_auresp_2.docx

# Peer review: Decision Letter 2

**DOI:** 10.1371/journal.pone.0360305.r005

**Authors:** Rajakumar Anbazhagan

4 Aug 2026

Dear Dr. Zhong,

Thank you for submitting your manuscript to PLOS ONE. After careful consideration, we feel that it has merit but does not fully meet PLOS ONE’s publication criteria as it currently stands. Therefore, we invite you to submit a revised version of the manuscript that addresses the points raised during the review process.

Please submit your revised manuscript by Sep 18 2026 11:59PM. If you will need more time than this to complete your revisions, please reply to this message or contact the journal office at plosone@plos.org. When you're ready to submit your revision, log on to https://www.editorialmanager.com/pone/ and select the 'Submissions Needing Revision' folder to locate your manuscript file.

- A letter that responds to each point raised by the academic editor and reviewer(s). You should upload this letter as a separate file labeled 'Response to Reviewers'.
- A marked-up copy of your manuscript that highlights changes made to the original version. You should upload this as a separate file labeled 'Revised Manuscript with Track Changes'.
- An unmarked version of your revised paper without tracked changes. You should upload this as a separate file labeled 'Manuscript'.

If you would like to make changes to your financial disclosure, please include your updated statement in your cover letter. Guidelines for resubmitting your figure files are available below the reviewer comments at the end of this letter.

If applicable, we recommend that you deposit your laboratory protocols in protocols.io to enhance the reproducibility of your results. Protocols.io assigns your protocol its own identifier (DOI) so that it can be cited independently in the future. For instructions see: https://journals.plos.org/plosone/s/submission-guidelines#loc-laboratory-protocols. Additionally, PLOS ONE offers an option for publishing peer-reviewed Lab Protocol articles, which describe protocols hosted on protocols.io. Read more information on sharing protocols at https://plos.org/protocols?utm_medium=editorial-email&utm_source=authorletters&utm_campaign=protocols.

As the corresponding author, your ORCID iD is verified in the submission system and will appear in the published article. PLOS supports the use of ORCID, and we encourage all coauthors to register for an ORCID iD and use it as well. Please encourage your coauthors to verify their ORCID iD within the submission system before final acceptance, as unverified ORCID iDs will not appear in the published article. *Only*  the individual author can complete the verification step; PLOS staff *cannot*  verify ORCID iDs on behalf of authors.

We look forward to receiving your revised manuscript.

Kind regards,

Rajakumar Anbazhagan

Academic Editor

PLOS One

Journal Requirements:

If the reviewer comments include a recommendation to cite specific previously published works, please review and evaluate these publications to determine whether they are relevant and should be cited. There is no requirement to cite these works unless the editor has indicated otherwise.

Please review your reference list to ensure that it is complete and correct. If you have cited papers that have been retracted, please include the rationale for doing so in the manuscript text, or remove these references and replace them with relevant current references. Any changes to the reference list should be mentioned in the rebuttal letter that accompanies your revised manuscript. If you need to cite a retracted article, indicate the article’s retracted status in the References list and also include a citation and full reference for the retraction notice.

[Note: HTML markup is below. Please do not edit.]

Reviewers' comments:

Reviewer's Responses to Questions

**Comments to the Author**

Reviewer #1: All comments have been addressed

Reviewer #2: All comments have been addressed

**********

2. Is the manuscript technically sound, and do the data support the conclusions?

Reviewer #1: Yes

Reviewer #2: Yes

**********

3. Has the statistical analysis been performed appropriately and rigorously?

Reviewer #1: Yes

Reviewer #2: Yes

**********

4. Have the authors made all data underlying the findings in their manuscript fully available?

The PLOS Data policy

Reviewer #1: Yes

Reviewer #2: Yes

**********

5. Is the manuscript presented in an intelligible fashion and written in standard English?

Reviewer #1: (No Response)

Reviewer #2: Yes

**********

Reviewer #1: (No Response)

Reviewer #2: Reviewer #2 (Second Revision)

I appreciate the authors' careful and thorough revision of the manuscript. The responses provided in the rebuttal letter are clear and comprehensive, and the corresponding changes have been appropriately incorporated into the revised manuscript. My previous concerns have been satisfactorily addressed, as summarized below.

1. WGCNA Minimum Module Size Inconsistency

Previous comment: The manuscript contained an inconsistency regarding the minimum module size used for WGCNA, reporting both 50 and 10 in different sections.

Assessment: The revised manuscript now consistently reports a minimum module size of 50 throughout both the Methods (Section 2.3) and Results (Section 3.2). The inconsistency has been fully resolved.

________________________________________

2. Control Group Terminology

Previous comment: References to "normal testis" or "normal controls" should be replaced with "OA controls" throughout the manuscript, figures, and tables to accurately reflect the comparator group.

Assessment: The manuscript now consistently refers to the comparator group as OA controls throughout the Methods, Results, and Discussion. The remaining reference to "normal human testicular tissues" in the Limitations section is appropriate because it specifically refers to truly fertile donor tissue that was unavailable for this study and is clearly distinguished from the OA controls used in the analysis. The authors also indicated that Figure 2H and Figure 5A were updated accordingly; these figure labels should simply be verified in the final production files. Overall, this concern has been satisfactorily addressed.

________________________________________

3. Translational Language in the Abstract

Previous comment: The Abstract contained language that overstated the translational implications of the study and should better reflect the exploratory nature of the findings.

Assessment: The authors have appropriately revised the Abstract. The study objective is now presented in a more exploratory manner, the description of the gene signature has been moderated, "diagnostic performance" has been replaced with "classification performance," and the discussion of drug repurposing has been revised to emphasize candidate drug-gene pairs requiring future experimental validation. The concluding statement has likewise been appropriately tempered. These revisions substantially improve the balance and scientific accuracy of the Abstract.

________________________________________

4. Machine Learning Evaluation

Previous comment: Confidence intervals or standard deviations for the reported AUC values should be provided to better characterize model performance during repeated cross-validation.

Assessment: The revised manuscript now reports results from 50 rounds of repeated five-fold cross-validation, including mean AUC, standard deviation, and 95% confidence intervals for all five machine learning classifiers in Supplementary Table S6. The accompanying explanation appropriately notes that these statistics reflect variability from repeated resampling rather than external validation, which is scientifically appropriate. This concern has been fully addressed.

________________________________________

Minor Editorial Observation

I noted one minor wording inconsistency in Section 3.3, where the manuscript states that "six machine learning algorithms" were evaluated, although only five algorithms are listed (Bagged Trees, Bayesian, Boruta, LASSO, and LVQ), consistent with the Methods, Abstract, and Figure 3 legend. This appears to be a typographical error and can be corrected during copyediting.

________________________________________

Overall Recommendation

The authors have addressed all of my previous comments thoroughly and appropriately. The revisions have improved the methodological clarity, terminology, and presentation of the manuscript, and I have no further scientific concerns. I recommend acceptance after correction of the minor editorial issue noted above.

**********

what does this mean?). If published, this will include your full peer review and any attached files.

If you choose “no”, your identity will remain anonymous but your review may still be made public.

**Do you want your identity to be public for this peer review?**  For information about this choice, including consent withdrawal, please see our Privacy Policy

Reviewer #1: No

Reviewer #2: No

**********

[NOTE: If reviewer comments were submitted as an attachment file, they will be attached to this email and accessible via the submission site. Please log into your account, locate the manuscript record, and check for the action link "View Attachments". If this link does not appear, there are no attachment files.]

To ensure your figures meet our technical requirements, please review our figure guidelines: https://journals.plos.org/plosone/s/figures

You may also use PLOS’s free figure tool, NAAS, to help you prepare publication quality figures: https://journals.plos.org/plosone/s/figures#loc-tools-for-figure-preparation.

NAAS will assess whether your figures meet our technical requirements by comparing each figure against our figure specifications.

Attachment Submitted filename: PONE-D-26-05936_Reviewer 2_Decision Letter_07282026.pdf

# Author comment: Author response to Decision Letter 3

**DOI:** 10.1371/journal.pone.0360305.r006

25 Aug 2026

We thank the reviewer for this careful observation. The description of “six machine learning algorithms” in Section 3.3 was a typographical error. We have corrected it to “five machine learning algorithms” in the revised manuscript to ensure full consistency with the Abstract, Methods, Figure 3 legend, and all relevant results throughout the text.

Attachment Submitted filename: Response_to_reviewers_auresp_3.docx

# Peer review: Decision Letter 3

**DOI:** 10.1371/journal.pone.0360305.r007

**Authors:** Rajakumar Anbazhagan

24 Sep 2026

Integrated Bioinformatics Analysis Identifies an Immune-Related Gene Signature Associated with Non-Obstructive Azoospermia

PONE-D-26-05936R3

Dear Dr. Zhong,

We’re pleased to inform you that your manuscript has been judged scientifically suitable for publication and will be formally accepted for publication once it meets all outstanding technical requirements.

Within one week, you’ll receive an e-mail detailing the required amendments. When these have been addressed, you’ll receive a formal acceptance letter and your manuscript will be scheduled for publication.

An invoice will be generated when your article is formally accepted. Please note, if your institution has a publishing partnership with PLOS and your article meets the relevant criteria, all or part of your publication costs will be covered. Please make sure your user information is up-to-date by logging into Editorial Manager at Editorial Manager® and clicking the ‘Update My Information' link at the top of the page. For questions related to billing, please contact billing support.

If your institution or institutions have a press office, please notify them about your upcoming paper to help maximize its impact. If they’ll be preparing press materials, please inform our press team as soon as possible -- no later than 48 hours after receiving the formal acceptance. Your manuscript will remain under strict press embargo until 2 pm Eastern Time on the date of publication. For more information, please contact onepress@plos.org.

Kind regards,

Academic Editor

PLOS One

Additional Editor Comments (optional):

Reviewers' comments:

Reviewer's Responses to Questions

**Comments to the Author**

Reviewer #1: All comments have been addressed

**********

2. Is the manuscript technically sound, and do the data support the conclusions?

Reviewer #1: Yes

**********

3. Has the statistical analysis been performed appropriately and rigorously?

Reviewer #1: Yes

**********

4. Have the authors made all data underlying the findings in their manuscript fully available?

The PLOS Data policy

Reviewer #1: Yes

**********

5. Is the manuscript presented in an intelligible fashion and written in standard English?

Reviewer #1: Yes

**********

Reviewer #1: The authors have substantially addressed my previous comments, particularly regarding the limitations of bulk transcriptomic analysis, the use of OA controls, the distinction between association and causality, and the exploratory nature of the drug–gene/docking analyses.

**********

what does this mean?). If published, this will include your full peer review and any attached files.

If you choose “no”, your identity will remain anonymous but your review may still be made public.

**Do you want your identity to be public for this peer review?**  For information about this choice, including consent withdrawal, please see our Privacy Policy

Reviewer #1: No

**********

# Editor report: Acceptance letter

**DOI:** 10.1371/journal.pone.0360305.r008

**Authors:** Rajakumar Anbazhagan

PONE-D-26-05936R3

PLOS One

Dear Dr. Zhong,

I'm pleased to inform you that your manuscript has been deemed suitable for publication in PLOS One. Congratulations! Your manuscript is now being handed over to our production team.

At this stage, our production department will prepare your paper for publication. This includes ensuring the following:

* All references, tables, and figures are properly cited

* All relevant supporting information is included in the manuscript submission,

* There are no issues that prevent the paper from being properly typeset

You will receive further instructions from the production team, including instructions on how to review your proof when it is ready. Please keep in mind that we are working through a large volume of accepted articles, so please give us a few days to review your paper and let you know the next and final steps.

Lastly, if your institution or institutions have a press office, please let them know about your upcoming paper now to help maximize its impact. If they'll be preparing press materials, please inform our press team within the next 48 hours. Your manuscript will remain under strict press embargo until 2 pm Eastern Time on the date of publication. For more information, please contact onepress@plos.org.

You will receive an invoice from PLOS for your publication fee after your manuscript has reached the completed accept phase. If you receive an email requesting payment before acceptance or for any other service, this may be a phishing scheme. Learn how to identify phishing emails and protect your accounts at https://explore.plos.org/phishing.

If we can help with anything else, please email us at customercare@plos.org.

Thank you for submitting your work to PLOS One and supporting open access.

Kind regards,

PLOS One Editorial Office Staff

on behalf of

Dr. Rajakumar Anbazhagan

Academic Editor

PLOS One
